# Structural basis of SARS-CoV-2 Omicron immune evasion and receptor engagement

**DOI:** 10.1126/science.abn8652

**Published:** 2022-01-27

**Authors:** Matthew McCallum, Nadine Czudnochowski, Laura E. Rosen, Samantha K. Zepeda, John E. Bowen, Alexandra C. Walls, Kevin Hauser, Anshu Joshi, Cameron Stewart, Josh R. Dillen, Abigail E. Powell, Tristan I. Croll, Jay Nix, Herbert W. Virgin, Davide Corti, Gyorgy Snell, David Veesler

**Affiliations:** ^1^ Department of Biochemistry, University of Washington, Seattle, WA 98195, USA.; ^2^ Vir Biotechnology, San Francisco, CA 94158, USA.; ^3^ Howard Hughes Medical Institute, University of Washington, Seattle, WA 98195, USA.; ^4^ Cambridge Institute for Medical Research, Department of Haematology, University of Cambridge, Cambridge, UK.; ^5^ Molecular Biology Consortium, Advanced Light Source, Lawrence Berkeley National Laboratory, Berkeley, CA, USA.; ​^6^ Department of Pathology and Immunology, Washington University School of Medicine, St. Louis, MO 63110, USA.; ^7^ Department of Internal Medicine, UT Southwestern Medical Center, Dallas, TX 75390, USA.; ^8^ Humabs Biomed SA, a subsidiary of Vir Biotechnology, 6500 Bellinzona, Switzerland.

## Abstract

The SARS-CoV-2 Omicron variant of concern evades antibody-mediated immunity that comes from vaccination or infection with earlier variants due to accumulation of numerous spike mutations. To understand the Omicron antigenic shift, we determined cryo-electron microscopy and X-ray crystal structures of the spike protein and the receptor-binding domain bound to the broadly neutralizing sarbecovirus monoclonal antibody (mAb) S309 (the parent mAb of sotrovimab) and to the human ACE2 receptor. We provide a blueprint for understanding the marked reduction of binding of other therapeutic mAbs that leads to dampened neutralizing activity. Remodeling of interactions between the Omicron receptor-binding domain and human ACE2 likely explains the enhanced affinity for the host receptor relative to the ancestral virus.

Although sequential COVID-19 waves have swept the world, no variants have accumulated mutations and mediated immune evasion to the extent observed for the SARS-CoV-2 Omicron (B.1.1.529) variant of concern (VOC). This variant was first identified late November 2021 in South Africa and was quickly designated a VOC by the World Health Organization (*1*). Omicron has spread worldwide at a rapid pace compared to previous SARS-CoV-2 variants (*2*, *3*). The Omicron spike (S) glycoprotein, which promotes viral entry into cells (*4*, *5*), harbors 37 residue mutations in the predominant haplotype relative to Wuhan-Hu-1 S (*4*), as compared to approximately 10 substitutions in both SARS-CoV-2 Alpha and Delta VOC (*2*, *6*). The Omicron receptor-binding domain (RBD) and the N-terminal domain (NTD) contain 15 and 11 mutations, respectively, which lead to severe dampening of plasma neutralizing activity in previously infected or vaccinated individuals (*7*–*11*). Although the Omicron RBD harbors 15 residue mutations, it binds to the human ACE2 entry receptor with high affinity while gaining the capacity to efficiently recognize mouse ACE2 (*7*). As a result of this antigenic shift, the only authorized or approved therapeutic monoclonal antibodies (mAbs) with neutralizing activity against Omicron are S309 (sotrovimab parent) and the COV2-2196/COV2-2130 cocktail (cilgavimab/tixagevimab parents). Even these mAbs experienced 2-3-fold and 12-200-fold reduced potency, respectively, using pseudovirus or authentic virus assays (*7*–*11*). This extent of evasion of humoral responses has important consequences for therapy and prevention of both the current pandemic and future pandemics, underscoring the necessity of defining the molecular mechanisms of these changes.

To provide a structural framework for the observed Omicron immune evasion and altered receptor recognition, we determined cryoEM structures of the prefusion-stabilized SARS-CoV-2 Omicron S ectodomain trimer bound to S309 and S2L20 (NTD-specific mAb) Fab fragments (Fig. 1, fig. S1, and table S1) and the X-ray crystal structure of the Omicron RBD in complex with human ACE2 and the Fab fragments of S309 and S304 at 2.85 Å resolution (table S2). S309 recognizes antigenic site IV (*12*) whereas S304 binds to site IIc (*13*) and was used to assist crystallization. Furthermore, we evaluated the binding of clinical mAbs to the Omicron RBD and S ectodomain trimer using surface plasmon resonance (SPR).

**Fig. 1.**
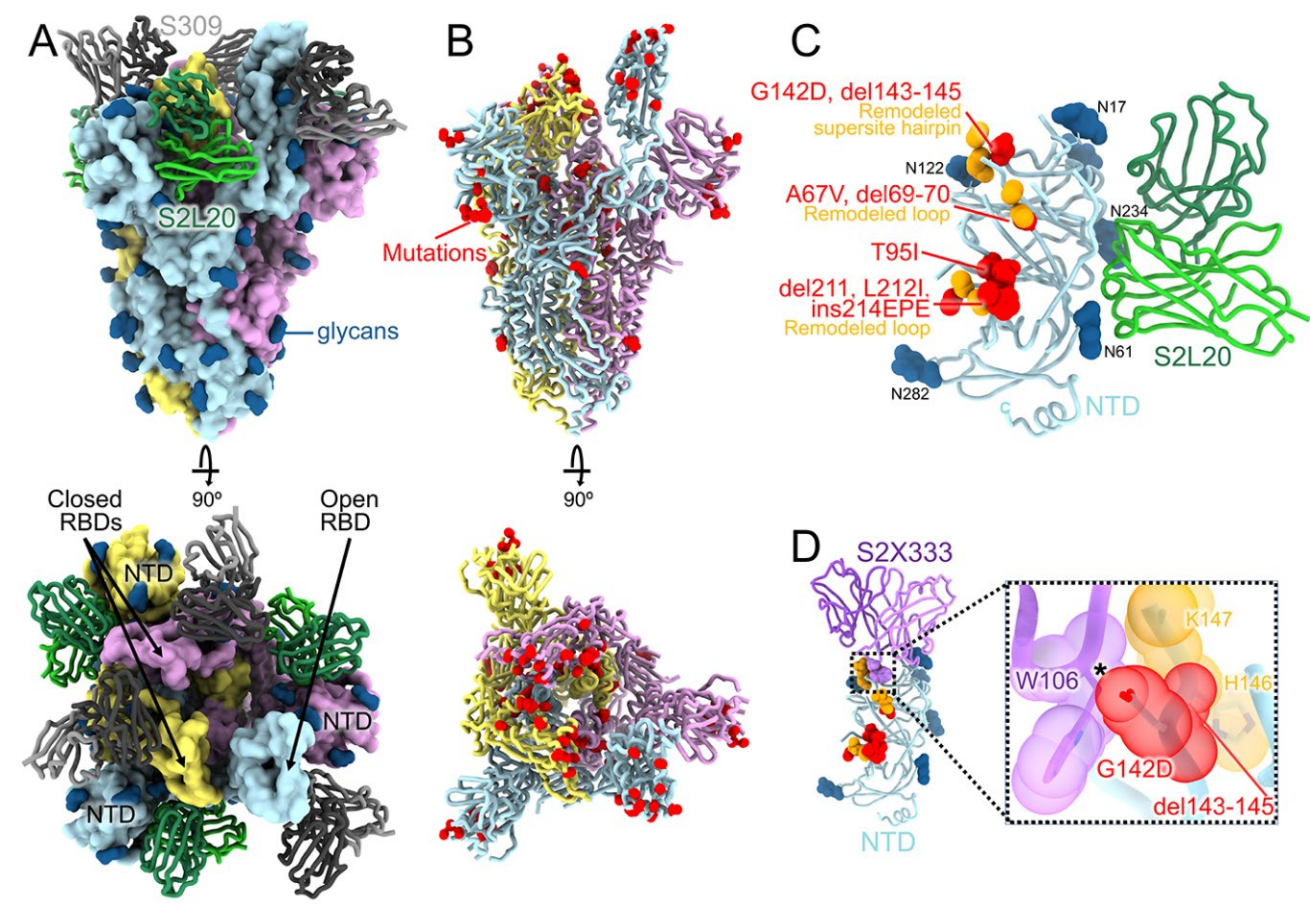
CryoEM structure of the SARS-CoV-2 Omicron S trimer reveals a remodeling of the NTD antigenic supersite. (**A**) Surface rendering in two orthogonal orientations of the Omicron S trimer with one open RBD bound to the S309 (grey) and S2L20 (green) Fabs shown as ribbons. The three S protomers are colored light blue, pink or gold. N-linked glycans are shown as dark blue surfaces. (**B**) Ribbon diagrams in two orthogonal orientations of the S trimer with one open RBD with Omicron residues mutated relative to Wuhan-Hu-1 shown as red spheres (except D614G which is not shown). (**C**) The S2L20-bound Omicron NTD with mutated, deleted, or inserted residues rendered or indicated as red spheres. Segments with notable structural changes are shown in orange and labeled. (**D**) Zoomed-in view of the Omicron NTD antigenic supersite overlaid with the S2X333 Fab (used here as an example of prototypical NTD neutralizing mAb (*22*)) highlighting the binding incompatibility; the modeled clash between S2X333 W106 and NTD G142D is indicated with an asterisk.

3D classification of the cryoEM data revealed the presence of two conformational states with one (45% of selected particles) or two (55% of selected particles) RBDs in the open conformation for which we determined structures at 3.1 Å and 3.2 Å resolution, respectively (Fig. 1, A and B, figs. S1 and S2, and table S1). The larger fraction of open RBDs, relative to the apo (*4*, *5*) and S309-bound (*12*) Wuhan-Hu-1 S ectodomain trimer structures, could result from the Omicron mutations, the prefusion-stabilizing mutations (*14*, *15*) or S2L20 binding. Focused classification and local refinement of the S309-bound RBD (domain B) and of the S2L20-bound NTD (domain A) were used to account for their conformational dynamics and improve local resolution of these regions to 3.0 and 3.3 Å resolution, respectively.

Whereas most VOC have only a few mutations beyond the NTD, RBD, and furin cleavage site regions, the Omicron spike harbors eight substitutions outside of these areas: T547K, H655Y, N764K, D796Y, N856K, Q954H, N969K, and L981F, which could all be modeled in the map (Fig. 1, A and B, and Fig. 2). Three of these mutations introduce inter-protomer electrostatic contacts between the S_2_ and S_1_ subunits: N764K binds Q314 (in domain D), S982 binds T547K (in domain C of protomers with closed RBDs), and N856K binds D568 and T572 (in domain C, the former residue is closer to N856K in protomers with closed RBDs) (Fig. 2) (*16*, *17*). Furthermore, N969K forms inter-protomer electrostatic contacts with Q755 and L981F improves intra-protomer hydrophobic packing in the pre-fusion conformation (Fig. 2). The latter mutation is close to the prefusion-stabilizing 2P mutations (K986P and V987P) used in all three vaccines deployed in the US (Fig. 2). Putatively enhanced interactions between the S_1_ and S_2_ subunits in Omicron S along with pre-fusion stabilization, and altered processing at the S_1_/S_2_ cleavage site due to the N679K and P681H mutations, might reduce S_1_ shedding, consistent with recent studies (*18*–*20*). Dampened S_1_ subunit shedding might enhance the effector function activity of vaccine- or infection-elicited Abs along with that of therapeutic mAbs (*21*) that retain affinity for Omicron S.

**Fig. 2.**
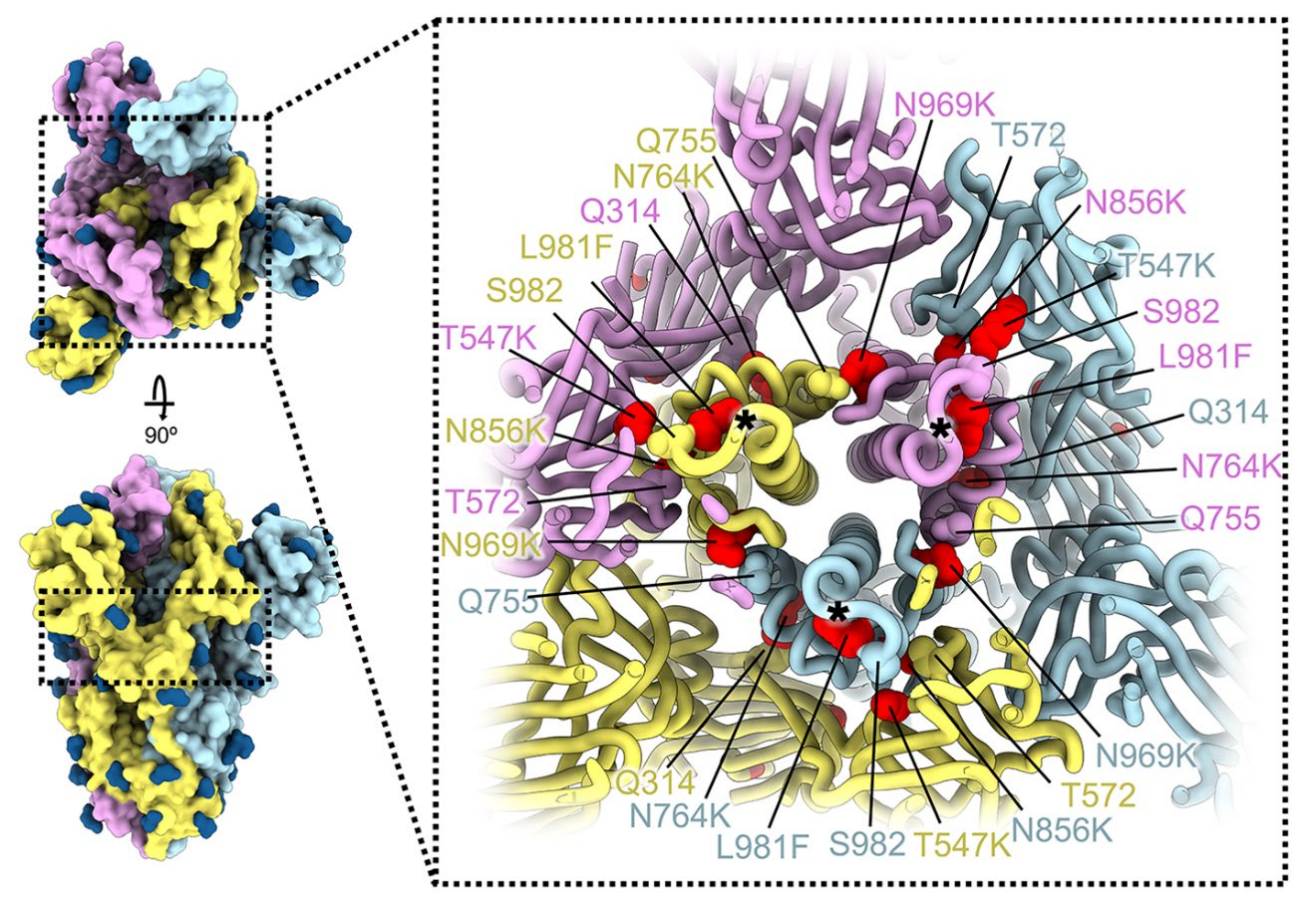
SARS-CoV-2 Omicron S mutations outside the NTD and RBD. Ribbon diagram showing a cross-section of the Omicron S glycoprotein (the location of this slice on the S trimer is indicated on the left). Mutated residues T547K, N764K, N856K, N969K, and L981F are shown as red spheres whereas the residues they contact are shown as spheres colored as the protomer they belong to. Black asterisks show the position of residues involved in the prefusion-stabilizing 2P mutations (K986P and V987P) used in all three vaccines deployed in the US. The three S protomers are colored light blue, pink or gold. N-linked glycans are shown as dark blue surfaces.

The Omicron NTD carries numerous mutations, deletions (del), and an insertion (ins) including A67V, del69-70, T95I, G142D, del143-145, del211, L212I, and ins214EPE (Fig. 1C and fig. S3). Many of these mutations have been described in previously emerged VOC: del69-70 was found in Alpha, T95I was present in Kappa and Iota, and G142D was present in Kappa and Delta. T95I, del211, L212I, and ins214EPE are outside the NTD antigenic supersite but in the vicinity of the epitope targeted by the P008_056 mAb, suggesting these mutations could putatively modulate recognition of similar mAbs or have another functional relevance. Although the region comprising del143-145 is weakly resolved in the map, it is expected to alter antibody recognition due to the introduced sequence register shift whereas G142D is incompatible with binding of several potent NTD neutralizing mAbs, such as S2X333, due to steric hindrance (Fig. 1D) (*2*, *22*). Moreover, del143-145 is reminiscent of the Alpha del144 which was also isolated as an escape mutation in the presence of mAb S2X333 and led to viral breakthrough in a hamster challenge model (*22*). These data suggest that G142D and del143-145 account for the observed SARS-CoV-2 Omicron evasion from neutralization mediated by a panel of NTD mAbs (*7*, *9*).

The RBD is the main target of plasma neutralizing activity in convalescent and vaccinated individuals and comprises several antigenic sites recognized by neutralizing Abs with a range of neutralization potencies and breadth (*12*, *13*, *21*, *23*–*36*) (Fig. 3A). Our structures provide a high-resolution blueprint of the residue substitutions found in this variant (Fig. 3B) and their impact on binding of clinical mAbs (Table 1). Several individual mutations or subsets of mutations occurring in the Omicron RBD have been reported previously to impact neutralizing antibody binding or neutralization (*37*). The K417N, G446S, S477N, T478K, E484A, Q493R, G496S, Q498R, N501Y and Y505H mutations are part of antigenic site I, which is immunodominant in previous variants (*13*, *24*). K417N, E484A, S477N, and Q493R would lead to loss of electrostatic interactions and steric clashes with REGN10933 whereas G446S would lead to steric clashes with REGN10987, consistent with the dampened binding to the Omicron RBD and S trimer (Fig. 3, C and D, fig. S3, and table S3) and with previous analyses of the impact of individual mutations on neutralization by each of these two mAbs (*9*, *38*–*40*). Moreover, N440K was reported to dampen REGN10987 neutralization severely (*9*). Reduced binding of the Omicron RBD to COV2-2196 and COV2-2130, relative to the Wuhan-Hu-1 RBD, likely results from the T478K (based on Delta S (*2*)), Q493R and putatively S477N for COV2-2196, as well as G446S and E484A for COV2-2130 (Fig. 3, E and F, fig. S4, and table S3). Integrating these data with neutralization assays suggests that although each point mutation only imparts a small reduction of COV2-2196- or COV2-2130-mediated neutralization (*9*), the constellation of Omicron mutations leads to more pronounced loss of activity (*7*–*11*). E484A abrogates electrostatic interactions with LY-CoV555 heavy and light chains, while Q493R would prevent binding through steric hindrance (Fig. 3G, fig. S4, and table S3), as supported by neutralization data (*9*). K417N is expected to negatively affect the constellation of electrostatic interactions formed between the Omicron RBD and LY-CoV16 heavy chain, thereby abolishing binding (Fig. 3H, fig. S4, and table S3) and neutralization of single mutant S pseudoviruses (*9*, *40*, *41*). Furthermore, S477N and Q493R have been shown to dampen binding of and neutralization mediated by LY-CoV16 (*9*, *41*). Finally, K417N, E484A and Q493R hinder CT-P59 engagement through a combination of steric hindrance and remodeling of electrostatic contacts, thereby preventing binding (Fig. 3I, fig. S4, and tables S1 and S3).

**Fig. 3.**
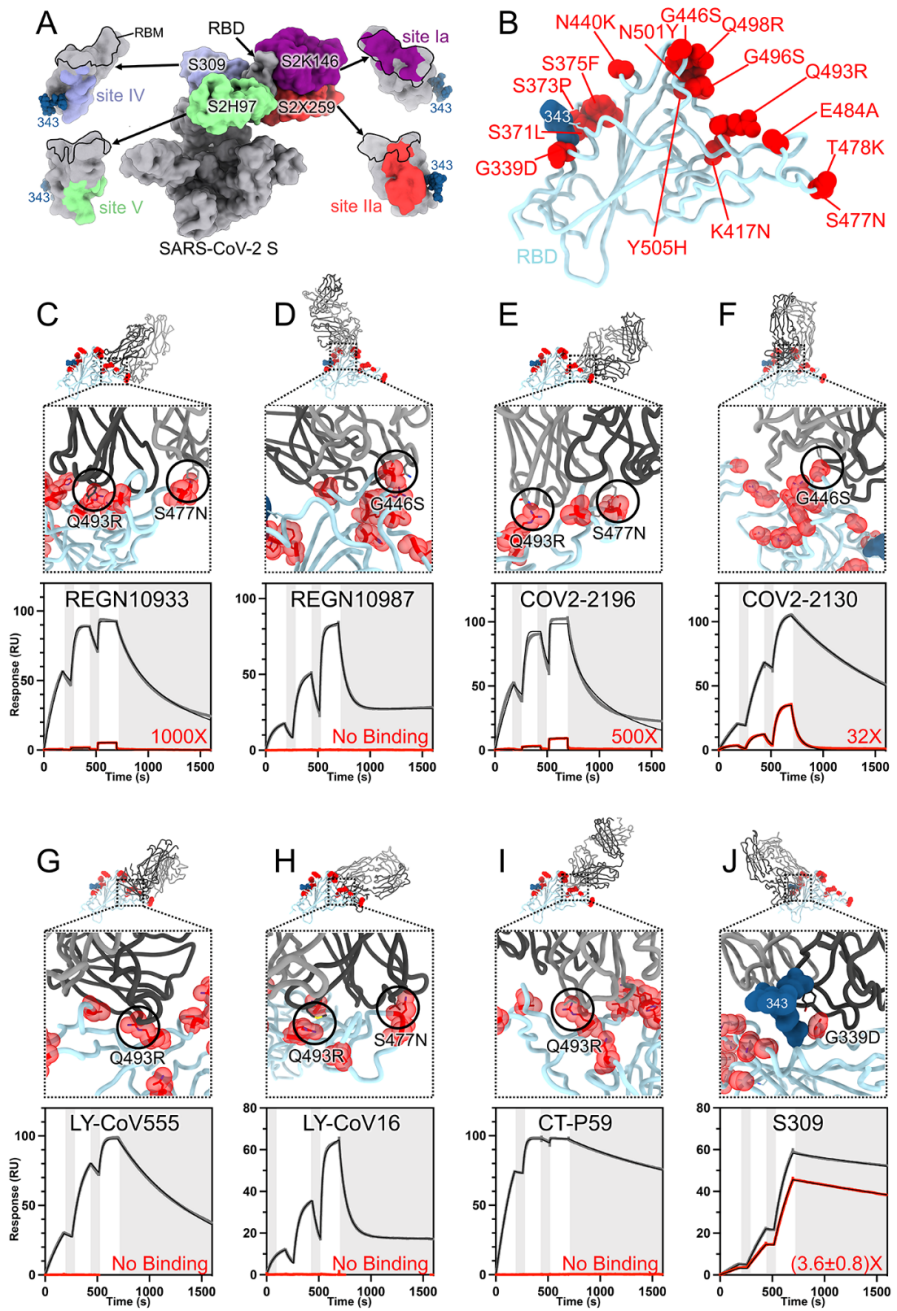
SARS-CoV-2 Omicron RBD mutations promote escape from a panel of clinical mAbs. (**A,** RBD antigenic map as determined elsewhere (*13*). (**B**) Ribbon diagram of the RBD crystal structure with residue mutated relative to the Wuhan-Hu-1 RBD shown as red spheres. The N343 glycan is rendered as blue spheres. (**C** to **J**) Zoomed-in view of the Omicron RBD (blue) superimposed on structures of clinical mAbs (grey) highlighting (black circles) selected residues that interfere with the mAbs: (C) REGN10933, (D) REGN10987, (E) COV2-2196, (F) COV2-2130, (G) LY-CoV555, (H) LY-CoV16, (I) CT-P59, and (J) S309 which does not clash with G339D. Panels A-I were rendered with the crystal structure whereas panel J was generated using the cryoEM model. Binding of the Wuhan-Hu-1 (gray line) or Omicron (red line) RBD to the corresponding mAb was evaluated using surface plasmon resonance (single-cycle kinetics) and is shown underneath each structural superimposition. White and gray stripes are association and dissociation phases, respectively. The black line is a fit to a kinetic model. The decrease in affinity between Wuhan-Hu-1 and Omicron binding is indicated in red. Results are consistent with IgG binding to S ectodomains (fig. S3).

**Table 1.** Omicron RBD mutations with a demonstrated (X) or expected (x) reduction of binding or neutralization and based on our structural analyses.

	REGN10933	REGN10987	COV2-2196	COV2-2130	LY-CoV555	LY-CoV016	CT-P59	S309	ADI-58125	Total GISAID counts**	Omicron counts	VOC, VOI, VUM harboring mutation
G339D										196,756	192,125	
S371L										182,692	179,486	
S373P										185,025	181,374	
S375F										184,990	181,461	
K417N	X					X	X			116,510	70,903	Beta, K417T in Gamma
N440K										92,338	79,859	
G446S		X		x						83,953	80,518	
S477N	x		X			x				262,216	187,081	
T478K			X							3,976,461	187,859	Delta
E484A	X			X	X		X			192,062	186,965	E484K in Beta, Gamma, Mu, Iota, Eta, Zeta, Theta; E484Q in Kappa
Q493R	X		X	x	X	x	X			191,484	188,353	
G496S							X			187,583	184,575	
Q498R							X			188,462	185,805	
N501Y										1,434,752	186,285	Alpha, Beta, Theta, N501K in Mu
Y505H									X*	188,250	185,491	
PDB ID	6XDG	6XDG	7L7D	7L7E	7KMG	7C01	7CM4	This study	n/a			

* For ADI-58125, the impact on binding of C, N, and S substitutions is shown at position Y505 based on mutagenesis studies [J. Belk *et al*., WO2021207597 - Compounds Specific to Coronavirus S Protein and Uses Thereof. Adagio Therapeutics, Inc. (2021)].

**As of January 9, 2022; excluded entries with >5% Ns.

The SARS-CoV-2 Omicron G339D and N440K mutations are within or nearby antigenic site IV, which is recognized by the S309 mAb (*12*). Nevertheless, S309 only experiences a 2 to 3-fold reduction of neutralizing activity against Omicron relative to Wuhan-Hu-1 pseudovirus or Washington-1 authentic virus (*7*, *9*–*11*). The lysine side chain introduced by the N440K substitution points away from the S309 epitope and does not affect binding. The aspartic acid side chain introduced by the G339D substitution does not interfere the S309 epitope although not all rotamers are compatible with mAb binding (fig. S2). This finding likely explains the similarly moderate reduction of S309 potency against the single G339D S mutant (*9*) or the full constellation of Omicron S mutations (*7*, *9*–*11*). The modest reduction of the Omicron RBD binding to S309 (Fig. 3J, fig. S4, and table S3) mirrors the 2-3-fold reduced neutralization potency of this VOC, relative to ancestral viruses, and concurs with deep-mutational scanning analysis of individual mutations on S309 recognition (*24*). Overall, the S309 binding mode remains unaltered by the Omicron mutations, including recognition of the N343 glycan (fig. S5).

The Omicron RBD is structurally similar to the Wuhan-Hu-1 RBD and both structures can be superimposed with an r.m.s.d. of 0.8Å over 183 aligned Cɑ residues (as compared to PDB 6m0j (*42*)). However, the region comprising residues 366 to 375, which harbors the S371L/S373P/S375F substitutions, deviates markedly from the conformation observed for the Wuhan-Hu-1 RBD, irrespective of the presence of bound linoleic acid (*4*, *42*, *43*). Although this region is weakly resolved in the cryoEM and X-ray structures, the conformation adopted in the latter structure is incompatible with binding of some cross-reactive site II mAbs such as S2X35, consistent with our observation of dampened binding (fig. S6). We therefore propose that these mutations participate in rendering this region of the RBD dynamic and mediate immune evasion from some site II mAbs.

We recently reported that the SARS-CoV-2 Omicron RBD binds human ACE2 with a ~2.4 fold enhanced affinity relative to the Wuhan-Hu-1 RBD (*7*). Our crystal structure of the human ACE2-bound Omicron RBD elucidates how the constellation of RBD mutations found in this VOC impact receptor recognition (Fig. 4A and table S2). The N501Y mutation alone enhances ACE2 binding to the RBD by a factor of 6 relative to the Wuhan-Hu-1 RBD, as reported for the Alpha variant (*6*), likely as a result of increased shape complementarity between the introduced tyrosine side chain and the ACE2 Y41 and K353 side chains (Fig. 4B). Omicron S residue Y501 and ACE2 residue Y41 form a T-shaped π–π stacking interaction, as previously observed for an N501Y-harboring S structure in complex with ACE2 (*44*). The K417N mutation dampens receptor recognition by about 3-fold (*2*, *6*, *39*, *45*) likely through loss of a salt bridge with ACE2 D30 (Fig. 4C). The Q493R and Q498R mutations introduce two new salt bridges with E35 and E38, respectively, replacing hydrogen bonds formed with the Wuhan-Hu-1 RBD, thereby remodeling the electrostatic interactions with ACE2 (Fig. 4D). Both of these individual mutations were reported to reduce ACE2 binding avidity slightly by deep-mutational scanning studies of the yeast-displayed SARS-CoV-2 RBD (*46*). Finally, S477N leads to formation of new hydrogen bonds between the introduced asparagine side chain and the ACE2 S19 backbone amine and carbonyl groups (Fig. 4E). Collectively, these mutations have a net enhancing effect on binding of the Omicron RBD to human ACE2, relative to Wuhan-Hu-1, suggesting that structural epistasis enables immune evasion while retaining efficient receptor engagement. The large number of Omicron mutations in the immunodominant receptor-binding motif likely explains a significant proportion of the loss of neutralization by convalescent and vaccine-elicited polyclonal antibodies, and is in line with the known plasticity of this subdomain (*24*).

**Fig. 4.**
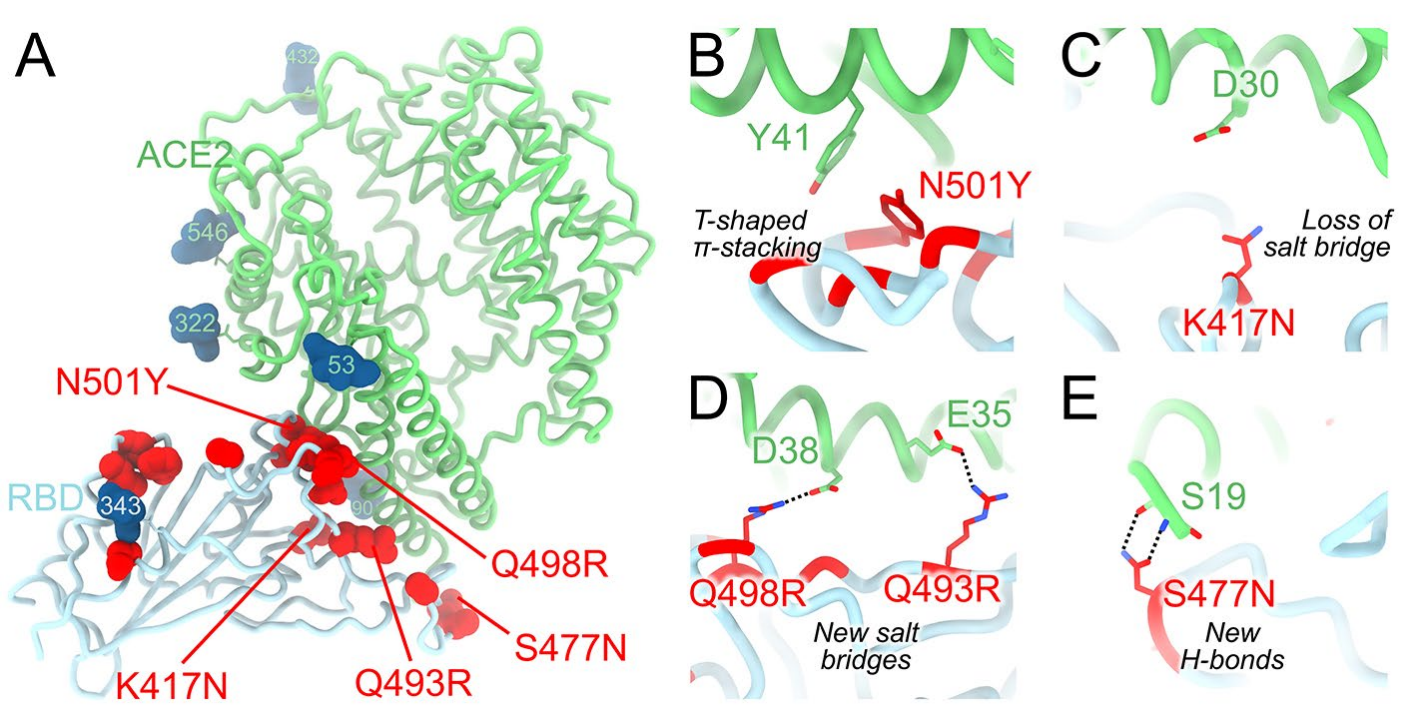
Molecular basis of human ACE2 recognition by the SARS-CoV-2 Omicron RBD. (**A**) Ribbon diagram of the crystal structure of the Omicron RBD in complex with the ACE2 ectodomain. The S309 and S304 Fab fragments are not shown for clarity. (**B** to **E**) Zoomed-in views of the RBD/ACE2 interface highlighting modulation of interactions due to introduction of the N501Y (B), K417N (C), Q493R/Q498R (D) and S477N (E) residue substitutions.

Although the N501Y mutation has been described previously to enable some SARS-CoV-2 VOC to infect and replicate in mice, the Alpha and Beta variant RBDs only weakly bound mouse ACE2 (*47*, *48*). The SARS-CoV-2 Omicron RBD, however, interacts more strongly with mouse ACE2 than the Alpha and Beta variant RBDs when evaluated side-by-side (fig. S7A) and can utilize mouse ACE2 as an entry receptor for S-mediated entry (*7*, *49*). We propose that the Q493R mutation plays a key role in enabling efficient mouse ACE2 binding, through formation of a new electrostatic interaction with the N31 side chain amide (K31 in human ACE2), as supported by in silico modeling based on our human ACE2-bound crystal structure (fig. S7B). These findings concur with the emergence and fixation of the Q493K RBD mutation upon serial passaging in mice to yield a mouse-adapted virus designated SARS-CoV-2 MA10 (*50*).

This work defines the molecular basis for the broad evasion of humoral immunity exhibited by SARS-CoV-2 Omicron and underscores the SARS-CoV-2 S mutational plasticity and the importance of targeting conserved epitopes in design and development of vaccines and therapeutics. The S309 mAb, which is the parent of sotrovimab, neutralizes Omicron with 2-3-fold reduced potency compared to Wuhan-Hu-1 or Washington-1, while the 7 other clinical mAbs or mAb cocktails experience reduction of neutralizing activity of 1-2 orders of magnitude or greater. Furthermore, some Omicron isolates (≈9%) harbor the R346K substitution which in conjunction with N440K (present in the main haplotype) leads to escape from C135 mAb-mediated neutralization (*25*, *51*). R346K does not affect S309 whether in isolation or in the context of the full constellation of Omicron mutations illustrating that mAbs targeting antigenic site IV can be differently affected by Omicron (*7*, *9*, *46*). Whereas C135 was identified from a SARS-CoV-2 convalescent donor (*25*), S309 was isolated from a subject who recovered from a SARS-CoV infection in 2003 (*12*); the latter strategy increased the likelihood of finding mAbs recognizing epitopes that are mutationally constrained throughout sarbecovirus evolution. The identification of broadly reactive mAbs that neutralize multiple distinct sarbecoviruses, including SARS-CoV-2 variants, pave the way for designing vaccines eliciting broad sarbecovirus immunity (*52*–*56*). These efforts offer hope that the same strategies that contribute to solving the current pandemic will prepare us for possible future sarbecovirus pandemics.

## Supplementary Materials

### This PDF file includes:

Materials and Methods Figs. S1 to S7 Tables S1 to S3 References (*57*–*77*)
