# Supplementary material for: Structural basis of SARS-CoV-2 Omicron immune evasion and receptor engagement
Source: Science. 2022 Jan 27;375(6583):eabn8652. doi: 10.1126/science.abn8652 (PMC9427005; doi:10.1126/science.abn8652)
Supplement: Supplementary file 1 — Materials and Methods Figs. S1 to S7 Tables S1 to S3 References (57–77) [file abn8652_sm.pdf]

Science
AAAS

Supplementary Materials for

Structural basis of SARS-CoV-2 Omicron immune evasion and receptor engagement

Matthew McCallum *et al*.

Corresponding authors: David Veesler, dveesler@uw.edu; Gyorgy Snell, gsnell@vir.bio

DOI: 10.1126/science.abn8652

This PDF file includes:

Materials and Methods
Figs. S1 to S7
Tables S1 to S3
References

Supplementary Materials for

Structural basis of SARS-CoV-2 Omicron immune evasion and receptor engagement

Matthew McCallum[1&], Nadine Czudnochowski[2&], Laura E. Rosen[2], Samantha K. Zepeda[1], John E. Bowen[1], Alexandra C. Walls[1,3], Kevin Hauser[2], Anshu Joshi[1], Cameron Stewart[1], Josh R. Dillen[2], Abigail E. Powell[2], Tristan I. Croll[4], Jay Nix[5], Herbert W. Virgin[2,6,7], Davide Corti[8], Gyorgy Snell[2*] and David Veesler[1,3*]

[1]Department of Biochemistry, University of Washington, Seattle, WA 98195, USA
[2]Vir Biotechnology, San Francisco, CA 94158, USA
[3]Howard Hughes Medical Institute, University of Washington, Seattle, WA 98195, USA
[4]Cambridge Institute for Medical Research, Department of Haematology, University of Cambridge, Cambridge, UK
[5]Molecular Biology Consortium, Advanced Light Source, Lawrence Berkeley National Laboratory, Berkeley, CA, USA
[6]Department of Pathology and Immunology, Washington University School of Medicine, Saint Louis MO 63110
[7]Department of Internal Medicine, UT Southwestern Medical Center, Dallas TX 75390
[8]Humabs Biomed SA, a subsidiary of Vir Biotechnology, 6500 Bellinzona, Switzerland

[&]These authors contributed equally to this work

*Correspondence: dveesler@uw.edu , gsnell@vir.bio

This PDF file includes:

Materials and Methods
Figs. S1 to S7
Tables S1 to S3

Materials and Methods

Production of recombinant spike glycoprotein

The SARS-CoV-2 S Omicron ectodomain contains the Omicron specific mutations A67V, Δ69-70, T95I, G142D/Δ143-145, Δ211/L212I, ins214EPE, G339D, S371L, S373P, S375F, K417N, N440K, G446S, S477N, T478K, E484A, Q493R, G496S, Q498R, N501Y, Y505H, T547K, D614G, H655Y, N679K, P681H, N764K, D796Y, N856K, Q954H, N969K, and L981F, in addition to VFLIP stabilizing mutations F817P, A892P, A899P, A942P, V987P, Y707C, and T883C. The SARS-CoV-2 S HexaPro ectodomain was previously described (*14*). Both S trimers were produced in 200 mL cultures of Expi293F Cells (ThermoFisher Scientific) grown in suspension using Expi293 Expression Medium (ThermoFisher Scientific) at 37°C in a humidified 8% CO2 incubator rotating at 130 rpm. Cells grown to a density of 2.5 million cells per mL were transfected using the ExpiFectamine 293 Transfection Kit (ThermoFisher Scientific) and cultivated for 2-4 days at which point the supernatant was harvested. S ectodomains were purified from clarified supernatants using a Cobalt affinity column (Cytiva, HiTrap TALON crude), washing with 20 column volumes of 20 mM Tris-HCl pH 8.0 and 150 mM NaCl or 20 mM sodium phosphate pH 8 and 100 mM NaCl and eluted with a gradient of 500 mM imidazole. The S ectodomain was then concentrated using a 100 kDa centrifugal filter (Amicon Ultra 0.5 mL centrifugal filters, MilliporeSigma), residual imidazole was washed away by consecutive dilutions in the centrifugal filter unit with 20 mM Tris-HCl pH 8.0 and 150 mM NaCl, and finally concentrated to 1 mg/mL before use immediately after purification. The various RBDs used for BLI were based on a previously reported construct (*4*) and produced and biotinylated as previously described (*2*, *6*, *46*).

CryoEM sample preparation and data collection

100 μL of 1 mg/mL SARS-CoV-2 S B.1.1.529 ectodomain was incubated with 40 μl 3.4 mg/mL S309 Fab for 10 min at 37°C in 150 mM NaCl and 20 mM Tris-HCl pH 8 and then 2.2 μL of 67 mg/mL S2L20 Fab was added and the mixture was incubated for 15 min at 37°C. Unbound Fab was then washed away with six consecutive dilutions in 400 μL of 20 mM Tris-HCl pH 8.0 and 150 mM NaCl over a 100 kDa centrifugal filter (Amicon Ultra 0.5 mL centrifugal filters, MilliporeSigma). The complex was concentrated to 3.5 mg/mL and 3 μL was immediately applied onto a freshly glow discharged 2.0/2.0 UltraFoil grid (84) (200 mesh), plunge frozen using a vitrobot MarkIV (ThermoFisher Scientific) using a blot force of −1 and 6.0 s blot time at 100% humidity and 23°C.

Data were acquired using the Leginon software (*57*) to control a FEI Titan Krios transmission electron microscope equipped with a Gatan K3 direct detector and operated at 300 kV with a Gatan Quantum GIF energy filter. The dose rate was adjusted to 3.75 counts/super- resolution pixel/s, and each movie was acquired in 75 frames of 40 ms with a pixel size of 0.843 Å and a defocus range comprised between −0.2 and −2.0 μm.

CryoEM data processing

Movie frame alignment, estimation of the microscope contrast-transfer function parameters, particle picking and extraction (with a downsampled pixel size of 1.686 Å and box size of 256 pixels^2) were carried out using Warp (*58*). Reference-free 2D classification was performed using cryoSPARC (*59*) to select well-defined particle images. 3D classification with 50 iterations each (angular sampling 7.5° for 25 iterations and 1.8° with local search for 25 iterations) were carried

out using Relion without imposing symmetry (*60*, *61*). 3D refinements were carried out using non-uniform refinement in cryoSPARC (*62*) before particle images were subjected to Bayesian polishing using Relion (*63*) during which particles were re-extracted with a box size of 512 Å at a pixel size of 0.843 Å. Next, 86 optics groups were defined based on the beam tilt angle used for data collection. Another round of non-uniform refinement in cryoSPARC was then performed concurrently with global and per-particle defocus refinement. For focused classification of the NTD, particles were symmetry-expanded and 3D classified in Relion without alignment using a mask that encompasses the NTD and the S2L20 VH/VL region. Focused classification of the RBD and the S309 VH/VL region was done on one of the two closed RBDs found in the asymmetric reconstruction with a single RBD open as described for the NTD/S2L20 but without symmetry expansion. Particles in well-formed 3D classes were then used for local refinement in cryoSPARC. Reported resolutions are based on the gold-standard Fourier shell correlation of 0.143 criterion and Fourier shell correlation curves were corrected for the effects of soft masking by high-resolution noise substitution (*64*, *65*).

CryoEM model building and analysis

UCSF Chimera (*66*) and Coot (*67*) were used to fit atomic models of S2L20, S309, and SARS-CoV-2 S (PDB 7SOB) into the cryo-EM maps. The model was then refined and rebuilt into the map using Coot (*67*), Rosetta (*68*, *69*), Phenix (*70*), and ISOLDE (*71*). Model validation and analysis used MolProbity (*72*), EMRinger (*73*), Phenix (*70*), and Privateer (*74*). Figures were generated using UCSF ChimeraX (*75*).

Monoclonal antibodies

Antibody VH and VL sequences for mAbs COV2-2130 (PDB ID 7L7E), COV2-2196 (PDB ID 7L7E, 7L7D), REGN10933 (PDB ID 6XDG), REGN10987 (PDB ID 6XDG) and ADI-58125 (PCT application WO2021207597, seq. IDs 22301 and 22311) were subcloned into heavy chain (human IgG1) and the corresponding light chain (human IgKappa, IgLambda) expression vectors respectively and produced in transiently transfected ExpiCHO-S cells (Thermo Fisher, #A29133) at 37°C and 8% CO2. Cells were transfected using ExpiFectamine. Transfected cells were supplemented 1 day after transfection with ExpiCHO Feed and ExpiFectamine CHO Enhancer. Cell culture supernatant was collected eight days after transfection and filtered through a 0.2 µm filter. Recombinant antibodies were affinity purified on an ÄKTA Xpress FPLC device using 5 mL HiTrap™ MabSelect™ PrismA columns followed by buffer exchange to Histidine buffer (20 mM Histidine, 8% sucrose, pH 6) using HiPrep 26/10 desalting columns. Antibody VH and VL sequences for LY-CoV555, LY-CoV016, and CT-P59 were obtained from PDB IDs 7KMG, 7C01 and 7CM4, respectively and mAbs were produced as recombinant IgG1 by ATUM. S309 was produced by WuXi Biologics (China). Recombinant S304 and S309 Fabs were produced by ATUM.

Recombinant protein production

SARS-CoV-2 RBD proteins for SPR binding assays (residues 328-531 of S protein from GenBank NC_045512.2 with N-terminal signal peptide and C-terminal thrombin cleavage site-TwinStrep-8xHis-tag) were expressed in Expi293F (Thermo Fisher Scientific) cells at 37°C and 8% CO2. Transfections were performed using the ExpiFectamine 293 Transfection Kit (Thermo Fisher Scientific). Cell culture supernatants were collected three days after transfection and supplemented with 10x PBS to a final concentration of 2.5x PBS (342.5 mM NaCl, 6.75 mM KCl and 29.75 mM

phosphates). RBDs were purified using cobalt-based immobilized metal affinity chromatography followed by buffer exchange into PBS using a HiPrep 26/10 desalting column (Cytiva) for Wuhan-Hu-1 protein, or a Superdex 200 Increase 10/300 GL column (Cytiva) for Omicron protein.
SARS-CoV-2 Omicron RBD for crystallization (residues 328-531, with N-terminal signal peptide and 'ETGT', and C-terminal 8xHis-tag) was expressed similarly as described above in the presence of 10 µM kifunensine. Cell culture supernatant was collected four days after transfection and supplemented with 10x PBS to a final concentration of 2.5x PBS. Protein was purified using a HiTrap TALON crude cartridge followed by size exclusion chromatography using a Superdex 200 Increase 10/300 GL column (Cytiva).
ACE2 for crystallization (residues 19-615 from Uniprot Q9BYF1 with a C-terminal thrombin cleavage site-TwinStrep-10xHis-GGG-tag, and N-terminal signal peptide) was expressed in ExpiCHO cells in the presence of 10 µM kifunensine at 37°C and 8% CO_2. Transfection was performed using the ExpiCHO transfection kit (Thermo Fisher Scientific). Cell culture supernatant was collected eight days after transfection and supplemented to a final concentration of 80 mM Tris-HCl pH 8.0, 100 mM NaCl, and then incubated with BioLock (IBA GmbH) solution. hACE2 was purified using a 5 mL StrepTrap HP column (Cytiva) followed by size exclusion chromatography using a Superdex 200 Increase 10/300 GL column (Cytiva) pre-equilibrated in PBS.

SPR binding measurements of IgG

SPR binding measurements were performed using a Biacore T200 instrument with a CM5 sensor chip covalently immobilized with Cytiva Human Antibody Capture Kit (RBD binding) or Cytiva His Capture Kit (S binding). Running buffer was Cytiva HBS-EP+ (pH 7.4). All measurements were performed at 25 °C. RBD analyte concentrations were 3.1, 12.5, and 50 nM, run as single-cycle kinetics. IgG analyte concentrations for S binding experiments were 11, 33, 100, and 300 nM, run as single-cycle kinetics. For RBD binding, double reference-subtracted data were fit using Biacore T200 Evaluation software (version 3.1) to a 1:1 binding model except two datasets (LY-CoV016 and REGN10987 binding to Wuhan-Hu-1 RBD) were fit to a Heterogeneous Ligand model ("biphasic fit") due to an artefactual kinetic phase with very slow dissociation that often arises when RBD is an analyte. For the Heterogeneous Ligand fits, the lower affinity of the two K_D values reported by the fit is taken as the K_D (the two K_D values are separated by at least two orders of magnitude). Binding of the Omicron RBD to mAbs has been carried out once (n=1) except for S309 which has been performed three times (n=3). Wuhan-Hu-1 RBD binding data are representative of at least two (n=2) for S309, CT-P59, LY-CoV555, LY-CoV16, REGN10987 and REGN10933 mAbs and once for all others (n=1). S binding data were evaluated qualitatively, rather than quantitatively, due to the avidity of the bivalent analyte precluding the use of a 1:1 binding model, and due to the anticipated differential impact of avidity on binding in the SPR experiment as compared to the biological context (e.g. S density and orientation on the CM5 matrix of the chip surface likely does not recapitulate S density and orientation on the surface of a virus or infected cell).

Crystallization, data collection, structure determination, and analysis

Prior to forming the SARS-CoV-2 Omicron RBD-ACE2-S304-S309 complex, recombinant SARS-CoV-2 Omicron RBD was digested with EndoH (New England Biolabs, 21 units/µg RBD) at 4°C overnight. Recombinant hACE2 protein was digested using EndoH (New England Biolabs, 25 units/µg ACE2) and thrombin (Sigma-Aldrich, 1 unit/75 µg ACE2) at 4°C overnight. RBD was

mixed with a 1.2-fold molar excess of EndoH deglycosylated hACE2, and a 1.1-fold molar excess of S304 Fab and S309 Fab. The complex was purified on a Superdex 200 10/300 GL column pre-equilibrated with 20 mM Tris-HCl pH 7.5, 150 mM NaCl. Crystals of the SARS-CoV-2 Omicron RBD-hACE2-S304-S309 complex were obtained at 20°C by sitting drop vapor diffusion. A total of 200 nL of the complex at 6.5 mg/mL were mixed with 200 nL mother liquor solution containing 0.1 M NDSB-256, 20% v/v ethylene glycol, 10% w/v PEG 8000, and 0.1 M Tris (base)/bicine pH 8.5.

Data were collected at the Molecular Biology Consortium beamline 4.2.2 at the Advanced Light Source synchrotron facility in Berkeley, CA and processed with the XDS software package yielding a final dataset of 2.85 Å in space group $P2_1$. The SARS-CoV-2 Omicron RBD-hACE2-S304-S309 complex structure was solved by molecular replacement using Phaser (*76*) from starting models consisting of RBD-S304-S309 (PDB: 7JX3) and hACE2 (PDB: 6m0j). Several subsequent rounds of model building and refinement were performed using Coot (*67*), ISOLDE (*71*), Refmac5 (*77*), Phenix (*70*) and MOE (https://www.chemcomp.com), to arrive at a final model of the quaternary complex.

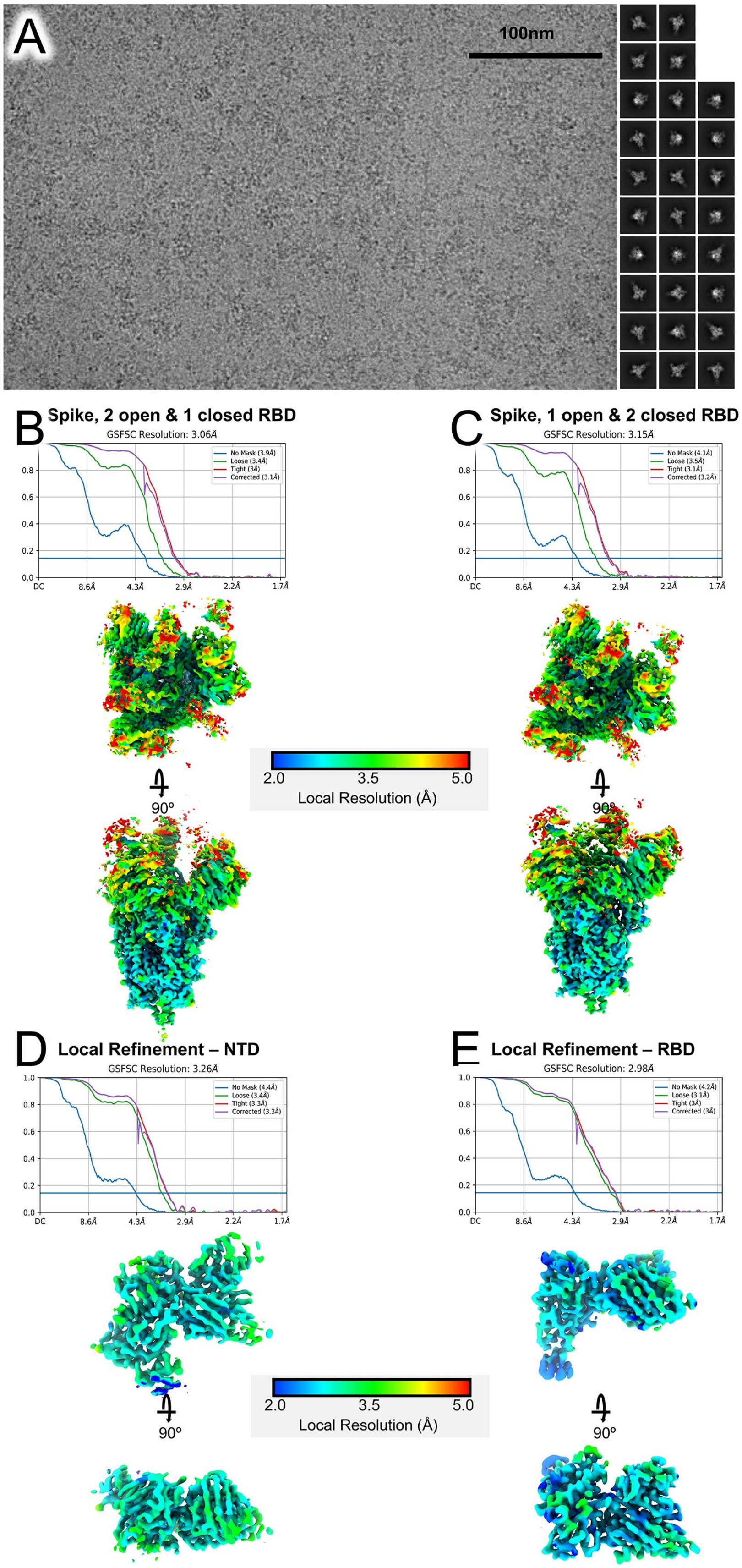
A
100nm
B
Spike, 2 open & 1 closed RBD
GSFSC Resolution: 3.06Å
No Mask (3.9Å)
Loose (3.4Å)
Tight (3Å)
Corrected (3.1Å)
C
Spike, 1 open & 2 closed RBD
GSFSC Resolution: 3.15Å
No Mask (4.1Å)
Loose (3.5Å)
Tight (3.1Å)
Corrected (3.2Å)
DC
8.6Å
4.3Å
2.9Å
2.2Å
1.7Å
2.0
3.5
5.0
Local Resolution (Å)
90º
D
Local Refinement – NTD
GSFSC Resolution: 3.26Å
No Mask (4.4Å)
Loose (3.4Å)
Tight (3.3Å)
Corrected (3.3Å)
E
Local Refinement – RBD
GSFSC Resolution: 2.98Å
No Mask (4.2Å)
Loose (3.1Å)
Tight (3Å)
Corrected (3Å)

Figure S1. CryoEM data processing and validation. (A) Representative electron micrograph (left, scale bar: 100 nm) and 2D class averages (right) are shown for the indicated particles embedded in vitreous ice. (**B-F**) Gold-standard Fourier shell correlation curves with the 0.143 cutoff indicated by a horizontal blue line (top) and unsharpened maps colored by local resolution calculated using cryoSPARC (bottom) for whole reconstructions (B, C, and D) and the locally refined reconstructions of NTD- or RBD-bound Fab variable domains (E and F).

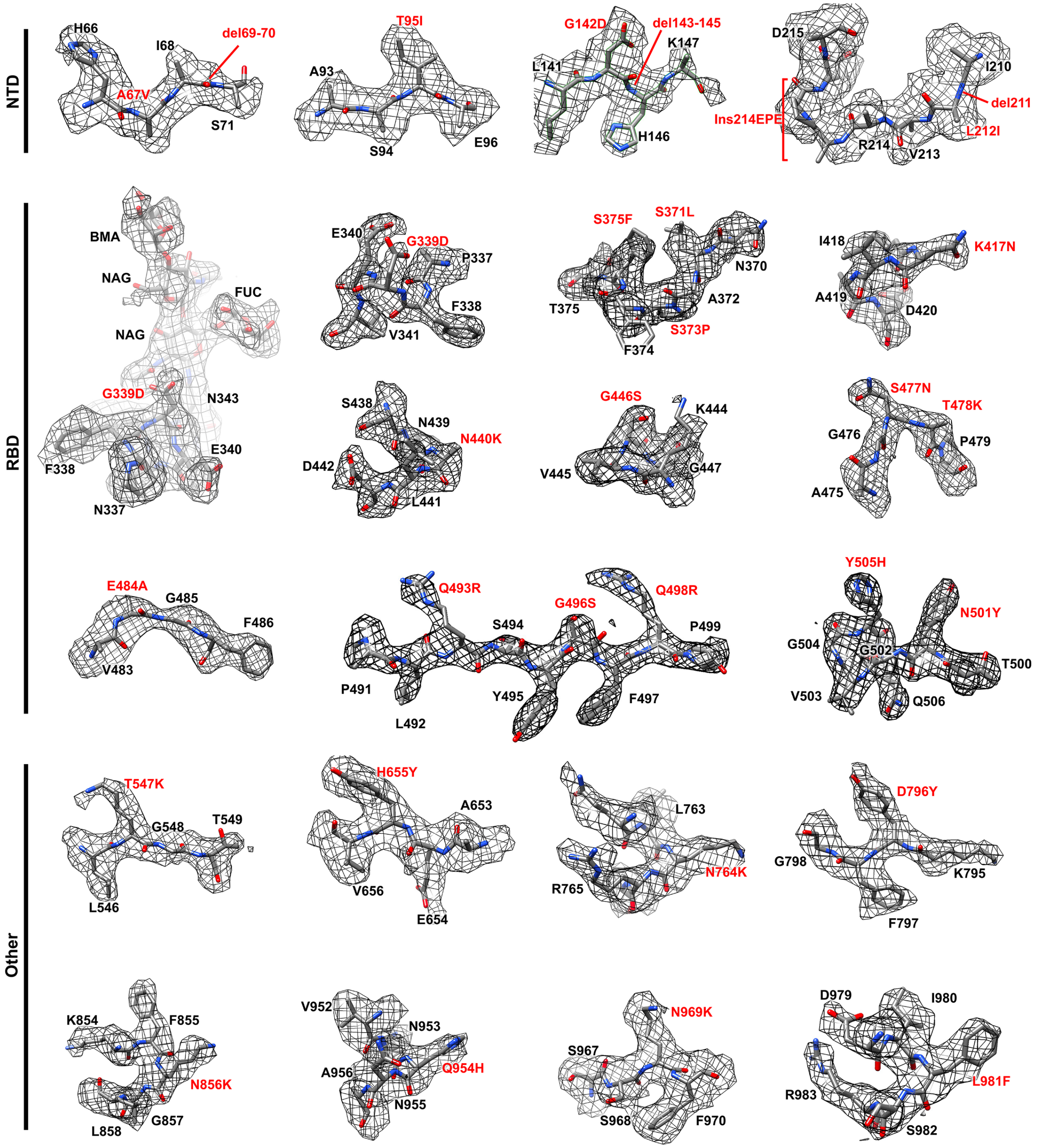

Figure S2. Density resolving the Omicron S mutations. The sharpened cryoEM map (NTD and

Other S regions) or 2Fo-Fc X-ray diffraction electron density (RBD) are rendered as mesh with the corresponding model shown as sticks (carbon: grey, nitrogen: blue, oxygen: red). The N343 glycan density corresponds to the cryoEM map.

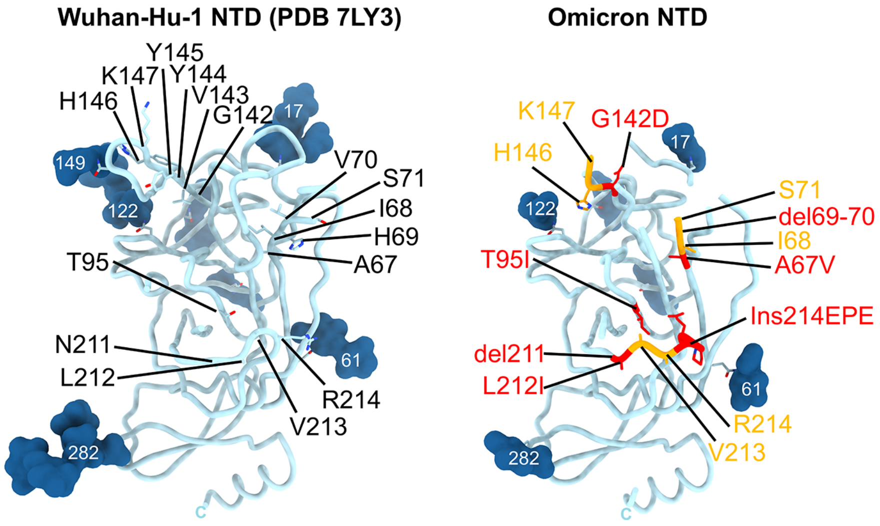

Figure S3. Comparison of Omicron and Wuhan-Hu-1 NTDs. Omicron mutated residues shown in red as sticks. The glycans are shown as dark blue surfaces.

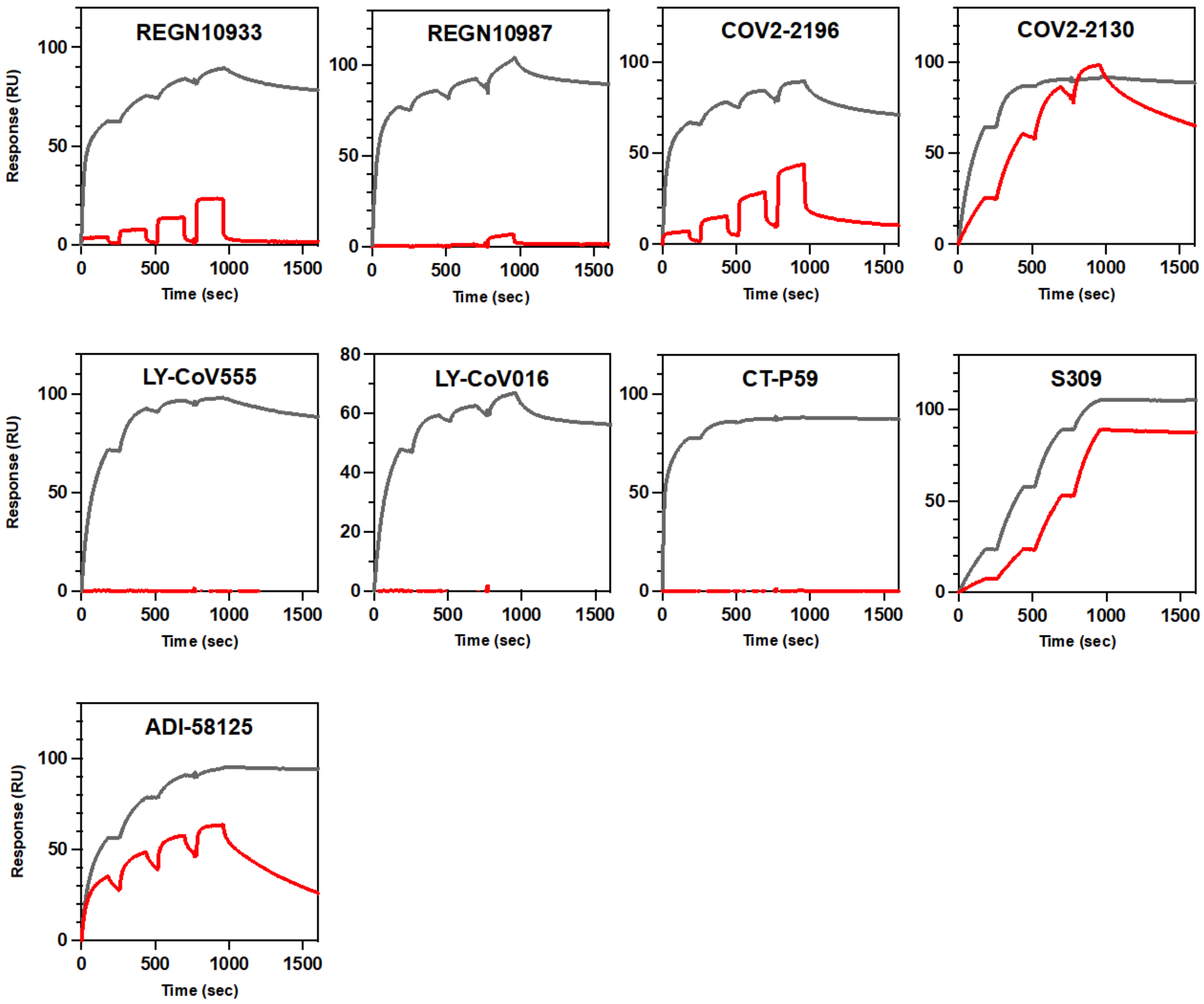

Fig S4. SARS-CoV-2 Omicron mutations promote escape from clinical mAb binding in the context of the full S ectodomain trimer. Binding of clinical-stage IgG to surface-immobilized S ectodomain, either Wuhan-Hu-1 (gray line) or Omicron (red line), was evaluated using single-cycle kinetics surface plasmon resonance (SPR) measurements. White and gray stripes are association and dissociation phases, respectively. Data reflect binding avidity due to the bivalent IgG in the mobile phase, and due to the required high density of S attached to the sensor chip matrix.

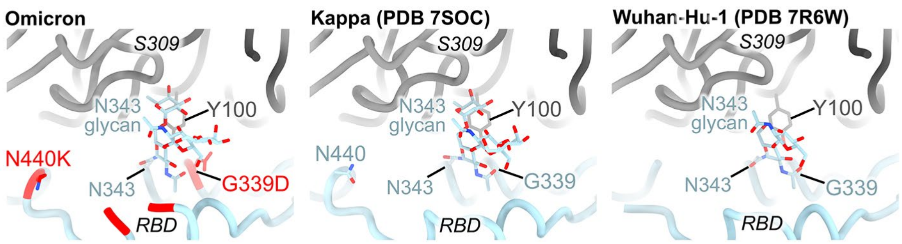

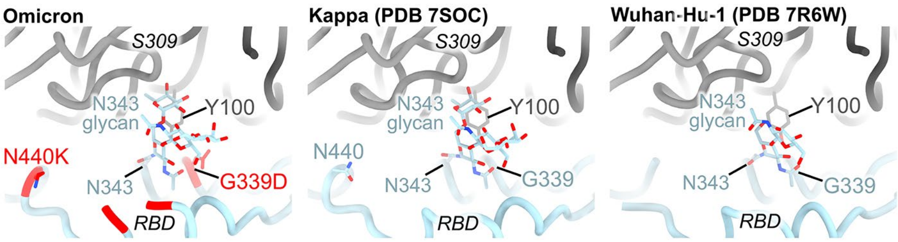

Figure S5. Structural basis for S309 binding to the Omicron, Kappa and Wuhan-Hu-1 RBD. Zoomed-in view of the cryoEM structure of the S309 Fab fragment (black/grey) bound to the Omicron, Kappa (*2*) or Wuhan-Hu-1 (*12*) RBD (blue ribbon) with Omicron mutated residues shown in red as sticks. The N343 glycan is shown as sticks.

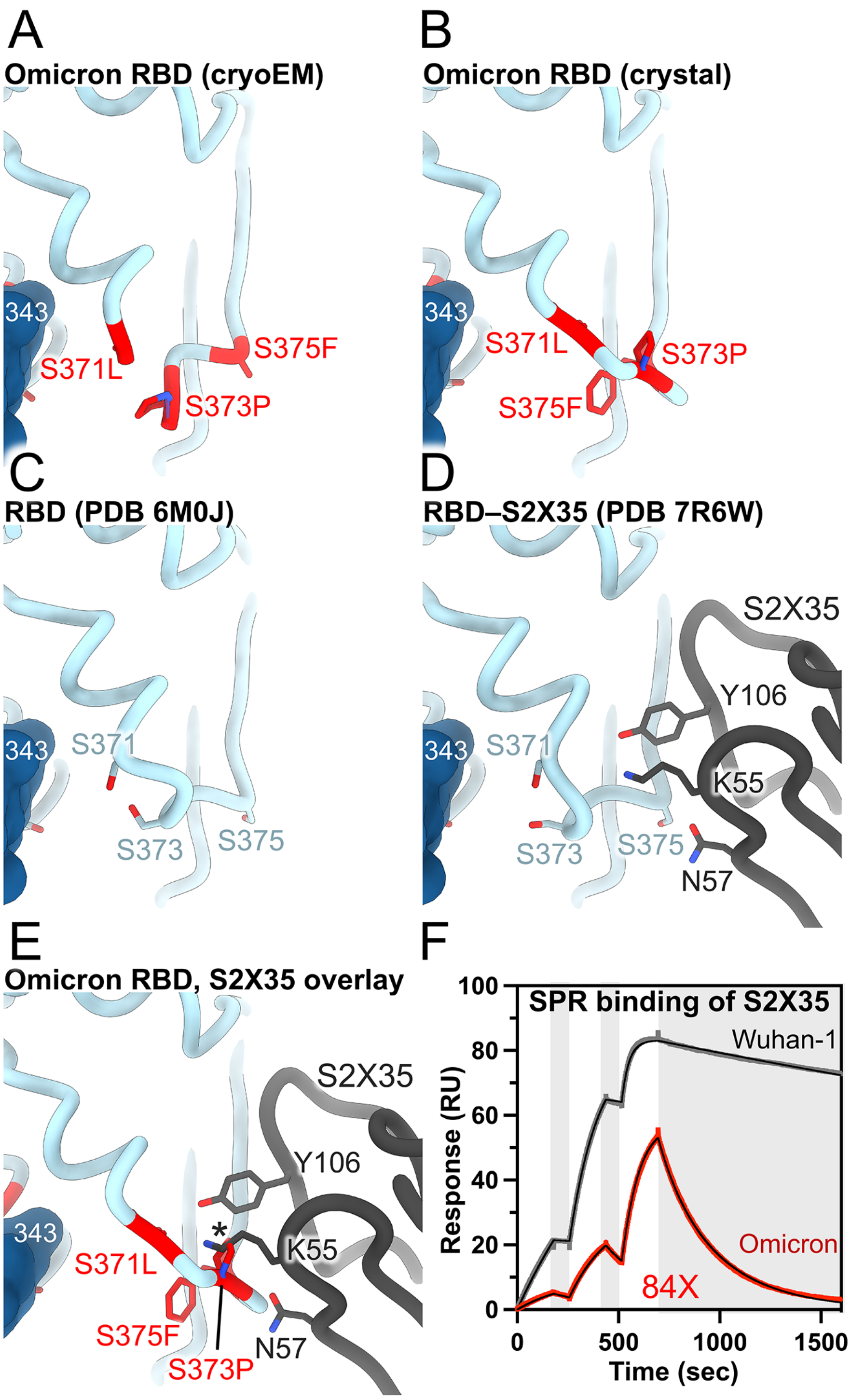

Fig S6. Comparison of the region comprising residues 366 to 375 across structures, and their impact on S2X35 binding. (A-D) Zoomed-in view of the Omicron RBD from the cryoEM structure (A) and crystal structure (B), as well as the Wuhan-1 RBD (C, PDB 6m0j) (*42*), and Wuhan-1 RBD bound to S2X35 (D, PDB 7r6w) (*24*). (**E**) Overlay of the RBD from (B) with S2X35 from (D) showing clashing between S373P and K55 (asterisk). (**F**) Binding of the Wuhan-Hu-1 (gray line) or Omicron (red line) RBD to S2X35 mAb was evaluated using surface

plasmon resonance (single-cycle kinetics). White and gray stripes are association and dissociation phases, respectively. The black line is a fit to a kinetic model. The decrease in affinity between Wuhan-Hu-1 and Omicron binding is indicated in red.

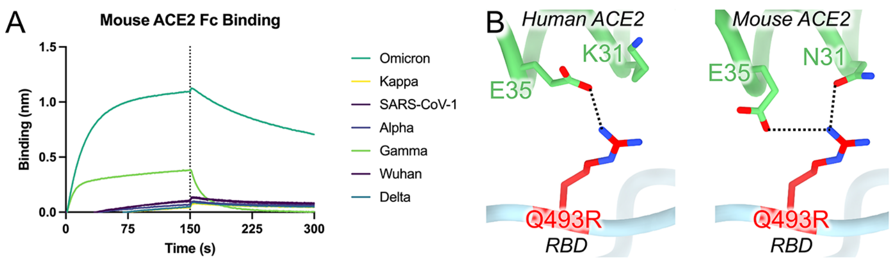

Fig S7. The SARS-CoV-2 Omicron RBD recognizes mouse ACE2 efficiently. A, Biolayer interferometry binding analysis of 1μM dimeric mouse ACE2-Fc to biotinylated SARS-CoV-2 variant RBDs immobilized at the surface of streptavidin (SA) biosensors. The vertical dashed lines indicate the transition between association and dissociation phases. **B,** In silico model of the interface between the Omicron RBD and mouse ACE2 (right, using PDB 7fdk) based on the crystal structure of the human ACE2-bound Omicron RBD (left).

Table S1: CryoEM data collection, processing and model refinement statistics.

	Omicron S<br>2 open 1 closed RBDs	Omicron S<br>2 closed/1 open RBDs		
**Data collection and processing**				
Magnification (nominal)	130,000	130,000		
Voltage (kV)	300	300		
Electron exposure (e^-/Å^2)	63	63		
Defocus range (μm)	0.3-2.0	0.3-2.0		
Pixel size (Å)	0.843	0.843		
Processing Type	Global	Global	Local (RBD)	Local (NTD)
Symmetry imposed	C1	C1	C1	C1
Initial particle images (no.)	482,905	482,905	224,478	224,478
Final particle images (no.)	55,316	74,826	155,712	94,770
Map resolution (Å)	3.2	3.1	3.0	3.3
FSC threshold	0.143	0.143	0.143	0.143
Map sharpening *B* factor (Å^2)	59	63	76	88
**Refinement**				
Initial model used (PDB code)	-	7SOB	7SOC	7SOD
Model resolution (Å)	-	3.2	3.3	3.4
FSC threshold	-	0.5	0.5	0.5
Model composition				
Nonhydrogen atoms	-	28,354	3,105	3,621
Protein residues	-	4443	408	475
Glycan residues	-	52	4	6
B factors (Å^2)				
Protein	-	46	20	24
Glycans	-	63	23	29
R.m.s. deviations				
Bond lengths (Å)	-	0.01	0.01	0.01
Bond angles (°)	-	1.4	1.1	1.1
**Validation**				
MolProbity score	-	0.98	1.0	0.9
Clashscore	-	1.49	1.7	0.7
Rotamer outliers (%)	-	0.17	0.0	0.3
Ramachandran plot				
Favored (%)	-	97.55	97	97
Allowed (%)	-	2.45	3	3
Outliers (%)	-	0	0	0
EMRinger score	-	3.91	5.2	5.5
**Data Availability**				
EMDB	EMD-25993	EMD-25992	EMD-25990	EMD-25991
PDB	-	7TM0	7TLY	7TLZ

Table S2: Crystallographic data collection and refinement statistics.

	Omicron RBD/ hACE2/S309/S304 PDB 7TN0
**Data collection**	
Space group	$P2_1$
Cell dimensions	
a, *b*, *c* (Å)	78.28, 183.65, 194.55
α, β, γ (°)	90.00, 95.98, 90.00
Resolution (Å)	48.91-2.85 (2.90-2.85)
R_{merge}	0.343 (2.676)
R_{pim}	0.153 (1.184)
$I / \sigma I$	5.6 (0.7)
CC(1/2)	0.975 (0.209)
Completeness (%)	96.7 (98.4)
Redundancy	5.8 (5.8)
**Refinement**	
Resolution (Å)	48.91-2.85
No. reflections	121127
R_{work} / R_{free}	23.03/26.79
No. atoms	
Protein	24486
Ligand/ion	221
Water	187
B-factors	
Protein	63.5
Ligand/ion	86.65
Water	42.64
R.m.s. deviations	
Bond lengths (Å)	0.003
Bond angles (°)	0.658
Ramachandran	
Favored (%)	97.6
Outliers (&)	0

*Values in parentheses are for highest-resolution shell.

Table S3: Kinetic parameters and fitting details of the SPR RBD binding measurements.

**1:1 Binding Model**						
**IgG**	**RBD**	**ka (1/Ms)**	**kd (1/s)**	**KD (M)**	**Rmax (RU)**	**Capture level (RU)**
S309	Wu-Hu-1	1.49E+05	1.32E-04	8.87E-10	73.77	210.5
S309	Omicron	7.15E+04	1.90E-04	2.67E-09	82.01	211.8
ADI-58125	Wu-Hu-1	1.15E+06	7.17E-08[a]	< 1E-11[a]	113.6	231
ADI-58125	Omicron	5.42E+06	0.2497	4.61E-08	83.81	230.3
COV2-2130	Wu-Hu-1	4.26E+05	8.38E-04	1.97E-09	109.5	251.7
COV2-2130	Omicron	9.30E+05	0.05897	6.34E-08	79.31	246.7
COV2-2196	Wu-Hu-1	1.14E+07	0.01244	1.09E-09	100.7	240.5
COV2-2196	Omicron	9.04E+05	0.441	4.88E-07	100[b]	239.7
CT-P59	Wu-Hu-1	5.54E+06	3.65E-04	6.60E-11	97.99	236.8
CT-P59	Omicron	No binding				236.9
LY-CoV016	Wu-Hu-1	See Biphasic fit results, below				196.1
LY-CoV016	Omicron	No binding				195
LY-CoV555	Wu-Hu-1	7.92E+05	0.001174	1.48E-09	100.7	242.1
LY-CoV555	Omicron	No binding				243.6
REGN10933	Wu-Hu-1	5.29E+06	0.003645	6.89E-10	93.53	260
REGN10933	Omicron	6.16E+05	0.5073	8.24E-07	94[b]	259.6
REGN10987	Wu-Hu-1	See Biphasic fit results, below				231.6
REGN10987	Omicron	No binding				231.5
S2X35	Wu-Hu-1	5.70E+05	1.53E-04	2.68E-10	83.6	230.1
S2X35	Omicron	2.43E+05	5.43E-03	2.24E-08	82.5	229.4

**Biphasic fit (Heterogeneous Ligand Model), binding to Wuhan-Hu-1 RBD[c]**							
**ka1 (1/Ms)**	**kd1 (1/s)**	**KD1 (M)**	**ka2 (1/Ms)**	**kd2 (1/s)**	**KD2 (M)**	**Rmax1 (RU)**	**Rmax2 (RU)**
LY-CoV016							
**1.24E+06**	**0.0172**	**1.38E-08**	1.79E+05	5.23E-05	2.91E-10	**59.66**	20.68
REGN10987							
**1.28E+06**	**0.0159**	**1.24E-08**	2.74E+05	1.02E-08	3.73E-14	**70.48**	28.6

[a]Measured dissociation is beyond the limit of detection. Reporting KD as an upper bound
[b]Rmax fixed to Rmax from Wuhan-Hu-1 fit (without constraint, Rmax from Omicron fit was implausibly much lower than Rmax for corresponding Wuhan-Hu-1 binding)
[c]Values for the kinetic phase attributed to real binding (vs an artifact) are highlighted in bold

References and Notes

1. R. Viana, S. Moyo, D. G. Amoako, H. Tegally, C. Scheepers, C. L. Althaus, U. J. Anyaneji, P. A. Bester, M. F. Boni, M. Chand, W. T. Choga, R. Colquhoun, M. Davids, K. Deforche, D. Doolabh, L. du Plessis, S. Engelbrecht, J. Everatt, J. Giandhari, M. Giovanetti, D. Hardie, V. Hill, N.-Y. Hsiao, A. Iranzadeh, A. Ismail, C. Joseph, R. Joseph, L. Koopile, S. L. Kosakovsky Pond, M. U. G. Kraemer, L. Kuate-Lere, O. Laguda-Akingba, O. Lesetedi-Mafoko, R. J. Lessells, S. Lockman, A. G. Lucaci, A. Maharaj, B. Mahlangu, T. Maponga, K. Mahlakwane, Z. Makatini, G. Marais, D. Maruapula, K. Masupu, M. Matshaba, S. Mayaphi, N. Mbhele, M. B. Mbulawa, A. Mendes, K. Mlisana, A. Mnguni, T. Mohale, M. Moir, K. Moruisi, M. Mosepele, G. Motsatsi, M. S. Motswaledi, T. Mphoyakgosi, N. Msomi, P. N. Mwangi, Y. Naidoo, N. Ntuli, M. Nyaga, L. Olubayo, S. Pillay, B. Radibe, Y. Ramphal, U. Ramphal, J. E. San, L. Scott, R. Shapiro, L. Singh, P. Smith-Lawrence, W. Stevens, A. Strydom, K. Subramoney, N. Tebeila, D. Tshiabuila, J. Tsui, S. van Wyk, S. Weaver, C. K. Wibmer, E. Wilkinson, N. Wolter, A. E. Zarebski, B. Zuze, D. Goedhals, W. Preiser, F. Treurnicht, M. Venter, C. Williamson, O. G. Pybus, J. Bhiman, A. Glass, D. P. Martin, A. Rambaut, S. Gaseitsiwe, A. von Gottberg, T. de Oliveira, Rapid epidemic expansion of the SARS-CoV-2 Omicron variant in southern Africa. *Nature* 10.1038/s41586-022-04411-y (2022). doi:10.1038/s41586-022-04411-y Medline

2. M. McCallum, A. C. Walls, K. R. Sprouse, J. E. Bowen, L. E. Rosen, H. V. Dang, A. De Marco, N. Franko, S. W. Tilles, J. Logue, M. C. Miranda, M. Ahlrichs, L. Carter, G. Snell, M. S. Pizzuto, H. Y. Chu, W. C. Van Voorhis, D. Corti, D. Veesler, Molecular basis of immune evasion by the Delta and Kappa SARS-CoV-2 variants. *Science* **374**, 1621–1626 (2021). doi:10.1126/science.abl8506 Medline

3. M. McCallum, J. Bassi, A. De Marco, A. Chen, A. C. Walls, J. Di Iulio, M. A. Tortorici, M.-J. Navarro, C. Silacci-Fregni, C. Saliba, K. R. Sprouse, M. Agostini, D. Pinto, K. Culap, S. Bianchi, S. Jaconi, E. Cameroni, J. E. Bowen, S. W. Tilles, M. S. Pizzuto, S. B. Guastalla, G. Bona, A. F. Pellanda, C. Garzoni, W. C. Van Voorhis, L. E. Rosen, G. Snell, A. Telenti, H. W. Virgin, L. Piccoli, D. Corti, D. Veesler, SARS-CoV-2 immune evasion by the B.1.427/B.1.429 variant of concern. *Science* **373**, 648–654 (2021). doi:10.1126/science.abi7994 Medline

4. A. C. Walls, Y. J. Park, M. A. Tortorici, A. Wall, A. T. McGuire, D. Veesler, Structure, Function, and Antigenicity of the SARS-CoV-2 Spike Glycoprotein. *Cell* **181**, 281–292.e6 (2020). doi:10.1016/j.cell.2020.02.058 Medline

5. D. Wrapp, N. Wang, K. S. Corbett, J. A. Goldsmith, C. L. Hsieh, O. Abiona, B. S. Graham, J. S. McLellan, Cryo-EM structure of the 2019-nCoV spike in the prefusion conformation. *Science* **367**, 1260–1263 (2020). doi:10.1126/science.abb2507 Medline

6. D. A. Collier, A. De Marco, I. A. T. M. Ferreira, B. Meng, R. Datir, A. C. Walls, S. A. Kemp, J. Bassi, D. Pinto, C. Silacci-Fregni, S. Bianchi, M. A. Tortorici, J. Bowen, K. Culap, S. Jaconi, E. Cameroni, G. Snell, M. S. Pizzuto, A. F. Pellanda, C. Garzoni, A. Riva, A. Elmer, N. Kingston, B. Graves, L. E. McCoy, K. G. C. Smith, J. R. Bradley, N. Temperton, L. Ceron-Gutierrez, G. Barcenas-Morales, W. Harvey, H. W. Virgin, A. Lanzavecchia, L. Piccoli, R. Doffinger, M. Wills, D. Veesler, D. Corti, R. K. Gupta, Sensitivity of SARS-CoV-2 B.1.1.7 to mRNA vaccine-elicited antibodies. *Nature* **593**, 136–141 (2021). doi:10.1038/s41586-021-03412-7

7. E. Cameroni, J. E. Bowen, L. E. Rosen, C. Saliba, S. K. Zepeda, K. Culap, D. Pinto, L. A. VanBlargan, A. De Marco, J. di Iulio, F. Zatta, H. Kaiser, J. Noack, N. Farhat, N. Czudnochowski, C. Havenar-Daughton, K. R. Sprouse, J. R. Dillen, A. E. Powell, A. Chen, C. Maher, L. Yin, D. Sun, L. Soriaga, J. Bassi, C. Silacci-Fregni, C. Gustafsson, N. M. Franko, J. Logue, N. T. Iqbal, I. Mazzitelli, J. Geffner, R. Grifantini, H. Chu, A. Gori, A. Riva, O. Giannini, A. Ceschi, P. Ferrari, P. E. Cippà, A. Franzetti-Pellanda, C. Garzoni, P. J. Halfmann, Y. Kawaoka, C. Hebner, L. A. Purcell, L. Piccoli, M. S. Pizzuto, A. C. Walls, M. S. Diamond, A. Telenti, H. W. Virgin, A. Lanzavecchia, G. Snell, D. Veesler, D. Corti, Broadly neutralizing antibodies overcome SARS-CoV-2 Omicron antigenic shift. *Nature* 10.1038/s41586-021-04386-2 (2021). doi:10.1038/s41586-021-04386-2 Medline

8. Y. Cao, J. Wang, F. Jian, T. Xiao, W. Song, A. Yisimayi, W. Huang, Q. Li, P. Wang, R. An, J. Wang, Y. Wang, X. Niu, S. Yang, H. Liang, H. Sun, T. Li, Y. Yu, Q. Cui, S. Liu, X. Yang, S. Du, Z. Zhang, X. Hao, F. Shao, R. Jin, X. Wang, J. Xiao, Y. Wang, X. S. Xie, Omicron escapes the majority of existing SARS-CoV-2 neutralizing antibodies. *Nature* 10.1038/s41586-021-04385-3 (2021). doi:10.1038/s41586-021-04385-3 Medline

9. L. Liu, S. Iketani, Y. Guo, J. F.-W. Chan, M. Wang, L. Liu, Y. Luo, H. Chu, Y. Huang, M. S. Nair, J. Yu, K. K.-H. Chik, T. T.-T. Yuen, C. Yoon, K. K.-W. To, H. Chen, M. T. Yin, M. E. Sobieszczyk, Y. Huang, H. H. Wang, Z. Sheng, K.-Y. Yuen, D. D. Ho, Striking antibody evasion manifested by the Omicron variant of SARS-CoV-2. *Nature* 10.1038/s41586-021-04388-0 (2021). doi:10.1038/s41586-021-04388-0 Medline

10. D. Planas, N. Saunders, P. Maes, F. Guivel-Benhassine, C. Planchais, J. Buchrieser, W.-H. Bolland, F. Porrot, I. Staropoli, F. Lemoine, H. Péré, D. Veyer, J. Puech, J. Rodary, G. Baele, S. Dellicour, J. Raymenants, S. Gorissen, C. Geenen, B. Vanmechelen, T. Wawina-Bokalanga, J. Martí-Carreras, L. Cuypers, A. Sève, L. Hocqueloux, T. Prazuck, F. Rey, E. Simon-Loriere, T. Bruel, H. Mouquet, E. André, O. Schwartz, Considerable escape of SARS-CoV-2 Omicron to antibody neutralization. *Nature* 10.1038/s41586-021-04389-z (2021). doi:10.1038/s41586-021-04389-z Medline

11. L. A. VanBlargan, J. M. Errico, P. J. Halfmann, S. J. Zost, J. E. Crowe Jr., L. A. Purcell, Y. Kawaoka, D. Corti, D. H. Fremont, M. S. Diamond, An infectious SARS-CoV-2

B.1.1.529 Omicron virus escapes neutralization by several therapeutic monoclonal antibodies. bioRxiv 472828 [preprint] (2021). doi:10.1101/2021.12.15.472828

12. D. Pinto, Y. J. Park, M. Beltramello, A. C. Walls, M. A. Tortorici, S. Bianchi, S. Jaconi, K. Culap, F. Zatta, A. De Marco, A. Peter, B. Guarino, R. Spreafico, E. Cameroni, J. B. Case, R. E. Chen, C. Havenar-Daughton, G. Snell, A. Telenti, H. W. Virgin, A. Lanzavecchia, M. S. Diamond, K. Fink, D. Veesler, D. Corti, Cross-neutralization of SARS-CoV-2 by a human monoclonal SARS-CoV antibody. *Nature* **583**, 290–295 (2020). doi:10.1038/s41586-020-2349-y Medline

13. L. Piccoli, Y. J. Park, M. A. Tortorici, N. Czudnochowski, A. C. Walls, M. Beltramello, C. Silacci-Fregni, D. Pinto, L. E. Rosen, J. E. Bowen, O. J. Acton, S. Jaconi, B. Guarino, A. Minola, F. Zatta, N. Sprugasci, J. Bassi, A. Peter, A. De Marco, J. C. Nix, F. Mele, S. Jovic, B. F. Rodriguez, S. V. Gupta, F. Jin, G. Piumatti, G. Lo Presti, A. F. Pellanda, M. Biggiogero, M. Tarkowski, M. S. Pizzuto, E. Cameroni, C. Havenar-Daughton, M. Smithey, D. Hong, V. Lepori, E. Albanese, A. Ceschi, E. Bernasconi, L. Elzi, P. Ferrari, C. Garzoni, A. Riva, G. Snell, F. Sallusto, K. Fink, H. W. Virgin, A. Lanzavecchia, D. Corti, D. Veesler, Mapping Neutralizing and Immunodominant Sites on the SARS-CoV-2 Spike Receptor-Binding Domain by Structure-Guided High-Resolution Serology. *Cell* **183**, 1024–1042.e21 (2020). doi:10.1016/j.cell.2020.09.037 Medline

14. C. L. Hsieh, J. A. Goldsmith, J. M. Schaub, A. M. DiVenere, H. C. Kuo, K. Javanmardi, K. C. Le, D. Wrapp, A. G. Lee, Y. Liu, C. W. Chou, P. O. Byrne, C. K. Hjorth, N. V. Johnson, J. Ludes-Meyers, A. W. Nguyen, J. Park, N. Wang, D. Amengor, J. J. Lavinder, G. C. Ippolito, J. A. Maynard, I. J. Finkelstein, J. S. McLellan, Structure-based design of prefusion-stabilized SARS-CoV-2 spikes. *Science* **369**, 1501–1505 (2020). doi:10.1126/science.abd0826 Medline

15. E. Olmedillas, C. J. Mann, W. Peng, Y. T. Wang, R. D. Avalos, Structure-based design of a highly stable, covalently-linked SARS-CoV-2 spike trimer with improved structural properties and immunogenicity. bioRxiv 441046 [preprint] (2021). doi:10.1101/2021.05.06.441046

16. A. C. Walls, M. A. Tortorici, B. J. Bosch, B. Frenz, P. J. M. Rottier, F. DiMaio, F. A. Rey, D. Veesler, Cryo-electron microscopy structure of a coronavirus spike glycoprotein trimer. *Nature* **531**, 114–117 (2016). doi:10.1038/nature16988 Medline

17. M. A. Tortorici, D. Veesler, Structural insights into coronavirus entry. *Adv. Virus Res.* **105**, 93–116 (2019). doi:10.1016/bs.aivir.2019.08.002 Medline

18. Z. Cong, J. P. Evans, P. Qu, J. Faraone, Y.-M. Zheng, C. Carlin, J. S. Bednash, T. Zhou, G. Lozanski, R. Mallampalli, L. J. Saif, E. M. Oltz, P. Mohler, K. Xu, R. J. Gumina, S.-L. Liu, Neutralization and Stability of SARS-CoV-2 Omicron Variant. bioRxiv 472934 [preprint] (2021). doi:10.1101/2021.12.16.472934

19. B. Meng, I. A. T. M. Ferreira, A. Abdullahi, S. A. Kemp, N. Goonawardane, G. Papa, S. Fatihi, O. J. Charles, D. A. Collier, J. Choi, J. H. Lee, P. Mlcochova, L. James, R. Doffinger, L. Thukral, K. Sato, R. K. Gupta, CITIID-NIHR BioResource COVID-19 Collaboration, The Genotype to Phenotype Japan (G2P-Japan) Consortium, SARS-CoV-2 Omicron spike mediated immune escape, infectivity and cell-cell fusion. bioRxiv 473248 [preprint] (2021). doi:10.1101/2021.12.17.473248

20. K. Sato, R. Suzuki, D. Yamasoba, I. Kimura, L. Wang, M. Kishimoto, J. Ito, Y. Morioka, N. Nao, H. Nasser, K. Uriu, Y. Kosugi, M. Tsuda, Y. Orba, M. Sasaki, R. Shimizu, R. Kawabata, K. Yoshimatsu, H. Asakura, M. Nagashima, K. Sadamasu, K. Yoshimura, H. Sawa, T. Ikeda, T. Irie, K. Matsuno, S. Tanaka, T. Fukuhara, Attenuated fusogenicity and pathogenicity of SARS-CoV-2 Omicron variant. Research Square 10.21203/rs.3.rs-1207670/v1 [preprint] (2022). doi:10.21203/rs.3.rs-1207670/v1

21. M. A. Tortorici, N. Czudnochowski, T. N. Starr, R. Marzi, A. C. Walls, F. Zatta, J. E. Bowen, S. Jaconi, J. Di Iulio, Z. Wang, A. De Marco, S. K. Zepeda, D. Pinto, Z. Liu, M. Beltramello, I. Bartha, M. P. Housley, F. A. Lempp, L. E. Rosen, E. Dellota Jr., H. Kaiser, M. Montiel-Ruiz, J. Zhou, A. Addetia, B. Guarino, K. Culap, N. Sprugasci, C. Saliba, E. Vetti, I. Giacchetto-Sasselli, C. S. Fregni, R. Abdelnabi, S. C. Foo, C. Havenar-Daughton, M. A. Schmid, F. Benigni, E. Cameroni, J. Neyts, A. Telenti, H. W. Virgin, S. P. J. Whelan, G. Snell, J. D. Bloom, D. Corti, D. Veesler, M. S. Pizzuto, Broad sarbecovirus neutralization by a human monoclonal antibody. *Nature* **597**, 103–108 (2021). doi:10.1038/s41586-021-03817-4 Medline

22. M. McCallum, A. De Marco, F. A. Lempp, M. A. Tortorici, D. Pinto, A. C. Walls, M. Beltramello, A. Chen, Z. Liu, F. Zatta, S. Zepeda, J. di Iulio, J. E. Bowen, M. Montiel-Ruiz, J. Zhou, L. E. Rosen, S. Bianchi, B. Guarino, C. S. Fregni, R. Abdelnabi, S. C. Foo, P. W. Rothlauf, L.-M. Bloyet, F. Benigni, E. Cameroni, J. Neyts, A. Riva, G. Snell, A. Telenti, S. P. J. Whelan, H. W. Virgin, D. Corti, M. S. Pizzuto, D. Veesler, N-terminal domain antigenic mapping reveals a site of vulnerability for SARS-CoV-2. *Cell* **184**, 2332–2347.e16 (2021). doi:10.1016/j.cell.2021.03.028 Medline

23. L. Stamatatos, J. Czartoski, Y.-H. Wan, L. J. Homad, V. Rubin, H. Glantz, M. Neradilek, E. Seydoux, M. F. Jennewein, A. J. MacCamy, J. Feng, G. Mize, S. C. De Rosa, A. Finzi, M. P. Lemos, K. W. Cohen, Z. Moodie, M. Juliana McElrath, A. T. McGuire, mRNA vaccination boosts cross-variant neutralizing antibodies elicited by SARS-CoV-2 infection. *Science* **372**, 1413–1418 (2021). doi:10.1126/science.abg9175

24. T. N. Starr, N. Czudnochowski, Z. Liu, F. Zatta, Y.-J. Park, A. Addetia, D. Pinto, M. Beltramello, P. Hernandez, A. J. Greaney, R. Marzi, W. G. Glass, I. Zhang, A. S. Dingens, J. E. Bowen, M. A. Tortorici, A. C. Walls, J. A. Wojcechowskyj, A. De Marco, L. E. Rosen, J. Zhou, M. Montiel-Ruiz, H. Kaiser, J. R. Dillen, H. Tucker, J. Bassi, C. Silacci-Fregni, M. P. Housley, J. di Iulio, G. Lombardo, M. Agostini, N. Sprugasci, K. Culap, S. Jaconi, M. Meury, E. Dellota Jr., R. Abdelnabi, S. C. Foo, E. Cameroni, S.

Stumpf, T. I. Croll, J. C. Nix, C. Havenar-Daughton, L. Piccoli, F. Benigni, J. Neyts, A. Telenti, F. A. Lempp, M. S. Pizzuto, J. D. Chodera, C. M. Hebner, H. W. Virgin, S. P. J. Whelan, D. Veesler, D. Corti, J. D. Bloom, G. Snell, SARS-CoV-2 RBD antibodies that maximize breadth and resistance to escape. *Nature* **597**, 97–102 (2021). doi:10.1038/s41586-021-03807-6 Medline

25. C. O. Barnes, C. A. Jette, M. E. Abernathy, K. A. Dam, S. R. Esswein, H. B. Gristick, A. G. Malyutin, N. G. Sharaf, K. E. Huey-Tubman, Y. E. Lee, D. F. Robbiani, M. C. Nussenzweig, A. P. West Jr., P. J. Bjorkman, SARS-CoV-2 neutralizing antibody structures inform therapeutic strategies. *Nature* **588**, 682–687 (2020). doi:10.1038/s41586-020-2852-1 Medline

26. C. A. Jette, A. A. Cohen, P. N. P. Gnanapragasam, F. Muecksch, Y. E. Lee, K. E. Huey-Tubman, F. Schmidt, T. Hatziioannou, P. D. Bieniasz, M. C. Nussenzweig, A. P. West Jr., J. R. Keeffe, P. J. Bjorkman, C. O. Barnes, Broad cross-reactivity across sarbecoviruses exhibited by a subset of COVID-19 donor-derived neutralizing antibodies. *Cell Rep.* **36**, 109760 (2021). doi:10.1016/j.celrep.2021.109760 Medline

27. C. O. Barnes, A. P. West Jr., K. E. Huey-Tubman, M. A. G. Hoffmann, N. G. Sharaf, P. R. Hoffman, N. Koranda, H. B. Gristick, C. Gaebler, F. Muecksch, J. C. C. Lorenzi, S. Finkin, T. Hägglöf, A. Hurley, K. G. Millard, Y. Weisblum, F. Schmidt, T. Hatziioannou, P. D. Bieniasz, M. Caskey, D. F. Robbiani, M. C. Nussenzweig, P. J. Bjorkman, Structures of Human Antibodies Bound to SARS-CoV-2 Spike Reveal Common Epitopes and Recurrent Features of Antibodies. *Cell* **182**, 828–842.e16 (2020). doi:10.1016/j.cell.2020.06.025 Medline

28. S. J. Zost, P. Gilchuk, J. B. Case, E. Binshtein, R. E. Chen, J. P. Nkolola, A. Schäfer, J. X. Reidy, A. Trivette, R. S. Nargi, R. E. Sutton, N. Suryadevara, D. R. Martinez, L. E. Williamson, E. C. Chen, T. Jones, S. Day, L. Myers, A. O. Hassan, N. M. Kafai, E. S. Winkler, J. M. Fox, S. Shrihari, B. K. Mueller, J. Meiler, A. Chandrashekar, N. B. Mercado, J. J. Steinhardt, K. Ren, Y. M. Loo, N. L. Kallewaard, B. T. McCune, S. P. Keeler, M. J. Holtzman, D. H. Barouch, L. E. Gralinski, R. S. Baric, L. B. Thackray, M. S. Diamond, R. H. Carnahan, J. E. Crowe Jr., Potently neutralizing and protective human antibodies against SARS-CoV-2. *Nature* **584**, 443–449 (2020). doi:10.1038/s41586-020-2548-6 Medline

29. D. R. Martinez, A. Schäfer, S. Gobeil, D. Li, G. De la Cruz, R. Parks, X. Lu, M. Barr, V. Stalls, K. Janowska, E. Beaudoin, K. Manne, K. Mansouri, R. J. Edwards, K. Cronin, B. Yount, K. Anasti, S. A. Montgomery, J. Tang, H. Golding, S. Shen, T. Zhou, P. D. Kwong, B. S. Graham, J. R. Mascola, D. C. Montefiori, S. M. Alam, G. D. Sempowski, S. Khurana, K. Wiehe, K. O. Saunders, P. Acharya, B. F. Haynes, R. S. Baric, A broadly cross-reactive antibody neutralizes and protects against sarbecovirus challenge in mice. *Sci. Transl. Med.* eabj7125 (2021). doi:10.1126/scitranslmed.abj7125 Medline

30. J. Dong, S. J. Zost, A. J. Greaney, T. N. Starr, A. S. Dingens, E. C. Chen, R. E. Chen, J. B. Case, R. E. Sutton, P. Gilchuk, J. Rodriguez, E. Armstrong, C. Gainza, R. S. Nargi, E. Binshtein, X. Xie, X. Zhang, P.-Y. Shi, J. Logue, S. Weston, M. E. McGrath, M. B. Frieman, T. Brady, K. M. Tuffy, H. Bright, Y.-M. Loo, P. M. McTamney, M. T. Esser, R. H. Carnahan, M. S. Diamond, J. D. Bloom, J. E. Crowe Jr., Genetic and structural basis for SARS-CoV-2 variant neutralization by a two-antibody cocktail. *Nat. Microbiol.* **6**, 1233–1244 (2021). doi:10.1038/s41564-021-00972-2 Medline

31. A. J. Greaney, A. N. Loes, L. E. Gentles, K. H. D. Crawford, T. N. Starr, K. D. Malone, H. Y. Chu, J. D. Bloom, Antibodies elicited by mRNA-1273 vaccination bind more broadly to the receptor binding domain than do those from SARS-CoV-2 infection. *Sci. Transl. Med.* **13**, eabi9915 (2021). doi:10.1126/scitranslmed.abi9915 Medline

32. J. Hansen, A. Baum, K. E. Pascal, V. Russo, S. Giordano, E. Wloga, B. O. Fulton, Y. Yan, K. Koon, K. Patel, K. M. Chung, A. Hermann, E. Ullman, J. Cruz, A. Rafique, T. Huang, J. Fairhurst, C. Libertiny, M. Malbec, W. Y. Lee, R. Welsh, G. Farr, S. Pennington, D. Deshpande, J. Cheng, A. Watty, P. Bouffard, R. Babb, N. Levenkova, C. Chen, B. Zhang, A. Romero Hernandez, K. Saotome, Y. Zhou, M. Franklin, S. Sivapalasingam, D. C. Lye, S. Weston, J. Logue, R. Haupt, M. Frieman, G. Chen, W. Olson, A. J. Murphy, N. Stahl, G. D. Yancopoulos, C. A. Kyratsous, Studies in humanized mice and convalescent humans yield a SARS-CoV-2 antibody cocktail. *Science* **369**, 1010–1014 (2020). doi:10.1126/science.abd0827 Medline

33. C. G. Rappazzo, L. V. Tse, C. I. Kaku, D. Wrapp, M. Sakharkar, D. Huang, L. M. Deveau, T. J. Yockachonis, A. S. Herbert, M. B. Battles, C. M. O'Brien, M. E. Brown, J. C. Geoghegan, J. Belk, L. Peng, L. Yang, Y. Hou, T. D. Scobey, D. R. Burton, D. Nemazee, J. M. Dye, J. E. Voss, B. M. Gunn, J. S. McLellan, R. S. Baric, L. E. Gralinski, L. M. Walker, Broad and potent activity against SARS-like viruses by an engineered human monoclonal antibody. *Science* **371**, 823–829 (2021). doi:10.1126/science.abf4830 Medline

34. A. Z. Wec, D. Wrapp, A. S. Herbert, D. P. Maurer, D. Haslwanter, M. Sakharkar, R. K. Jangra, M. E. Dieterle, A. Lilov, D. Huang, L. V. Tse, N. V. Johnson, C. L. Hsieh, N. Wang, J. H. Nett, E. Champney, I. Burnina, M. Brown, S. Lin, M. Sinclair, C. Johnson, S. Pudi, R. Bortz 3rd, A. S. Wirchnianski, E. Laudermilch, C. Florez, J. M. Fels, C. M. O'Brien, B. S. Graham, D. Nemazee, D. R. Burton, R. S. Baric, J. E. Voss, K. Chandran, J. M. Dye, J. S. McLellan, L. M. Walker, Broad neutralization of SARS-related viruses by human monoclonal antibodies. *Science* **369**, 731–736 (2020). doi:10.1126/science.abc7424 Medline

35. Y.-J. Park, A. De Marco, T. N. Starr, Z. Liu, D. Pinto, A. C. Walls, F. Zatta, S. K. Zepeda, J. E. Bowen, K. R. Sprouse, A. Joshi, M. Giurdanella, B. Guarino, J. Noack, R. Abdelnabi, S. C. Foo, L. E. Rosen, F. A. Lempp, F. Benigni, G. Snell, J. Neyts, S. P. J. Whelan, H. W. Virgin, J. D. Bloom, D. Corti, M. S. Pizzuto, D. Veesler, Antibody-mediated broad

sarbecovirus neutralization through ACE2 molecular mimicry. *Science* eabm8143 (2022). doi:10.1126/science.abm8143 Medline

36. J. E. Bowen, A. C. Walls, A. Joshi, K. R. Sprouse, C. Stewart, M. A. Tortorici, N. M. Franko, J. K. Logue, I. G. Mazzitelli, S. W. Tiles, K. Ahmed, A. Shariq, G. Snell, N. T. Iqbal, J. Geffner, A. Bandera, A. Gori, R. Grifantini, H. Y. Chu, W. C. Van Voorhis, D. Corti, D. Veesler, SARS-CoV-2 spike conformation determines plasma neutralizing activity. bioRxiv 473391 [preprint] (2021). doi:10.1101/2021.12.19.473391

37. D. Corti, L. A. Purcell, G. Snell, D. Veesler, Tackling COVID-19 with neutralizing monoclonal antibodies. *Cell* **184**, 3086–3108 (2021). doi:10.1016/j.cell.2021.05.005

38. T. Tada, B. M. Dcosta, M. I. Samanovic, R. S. Herati, A. Cornelius, H. Zhou, A. Vaill, W. Kazmierski, M. J. Mulligan, N. R. Landau, Convalescent-phase Sera and vaccine-elicited antibodies largely maintain neutralizing titer against global SARS-CoV-2 variant spikes. *mBio* **12**, e0069621 (2021). doi:10.1128/mBio.00696-21 Medline

39. M. Yuan, D. Huang, C. D. Lee, N. C. Wu, A. M. Jackson, X. Zhu, H. Liu, L. Peng, M. J. van Gils, R. W. Sanders, D. R. Burton, S. M. Reincke, H. Prüss, J. Kreye, D. Nemazee, A. B. Ward, I. A. Wilson, Structural and functional ramifications of antigenic drift in recent SARS-CoV-2 variants. *Science* **373**, 818–823 (2021). doi:10.1126/science.abh1139 Medline

40. P. Wang, M. S. Nair, L. Liu, S. Iketani, Y. Luo, Y. Guo, M. Wang, J. Yu, B. Zhang, P. D. Kwong, B. S. Graham, J. R. Mascola, J. Y. Chang, M. T. Yin, M. Sobieszczyk, C. A. Kyratsous, L. Shapiro, Z. Sheng, Y. Huang, D. D. Ho, Antibody resistance of SARS-CoV-2 variants B.1.351 and B.1.1.7. *Nature* **593**, 130–135 (2021). doi:10.1038/s41586-021-03398-2 Medline

41. T. N. Starr, A. J. Greaney, A. Addetia, W. W. Hannon, M. C. Choudhary, A. S. Dingens, J. Z. Li, J. D. Bloom, Prospective mapping of viral mutations that escape antibodies used to treat COVID-19. *Science* **371**, 850–854 (2021). doi:10.1126/science.abf9302 Medline

42. J. Lan, J. Ge, J. Yu, S. Shan, H. Zhou, S. Fan, Q. Zhang, X. Shi, Q. Wang, L. Zhang, X. Wang, Structure of the SARS-CoV-2 spike receptor-binding domain bound to the ACE2 receptor. *Nature* **581**, 215–220 (2020). doi:10.1038/s41586-020-2180-5 Medline

43. C. Toelzer, K. Gupta, S. K. N. Yadav, U. Borucu, A. D. Davidson, M. Kavanagh Williamson, D. K. Shoemark, F. Garzoni, O. Staufer, R. Milligan, J. Capin, A. J. Mulholland, J. Spatz, D. Fitzgerald, I. Berger, C. Schaffitzel, Free fatty acid binding pocket in the locked structure of SARS-CoV-2 spike protein. *Science* **370**, 725–730 (2020). doi:10.1126/science.abd3255 Medline

44. X. Zhu, D. Mannar, S. S. Srivastava, A. M. Berezuk, J.-P. Demers, J. W. Saville, K. Leopold, W. Li, D. S. Dimitrov, K. S. Tuttle, S. Zhou, S. Chittori, S. Subramaniam, Cryo-electron microscopy structures of the N501Y SARS-CoV-2 spike protein in complex with ACE2

and 2 potent neutralizing antibodies. *PLOS Biol.* **19**, e3001237 (2021). doi:10.1371/journal.pbio.3001237 Medline

45. E. C. Thomson, L. E. Rosen, J. G. Shepherd, R. Spreafico, A. da Silva Filipe, J. A. Wojcechowskyj, C. Davis, L. Piccoli, D. J. Pascall, J. Dillen, S. Lytras, N. Czudnochowski, R. Shah, M. Meury, N. Jesudason, A. De Marco, K. Li, J. Bassi, A. O'Toole, D. Pinto, R. M. Colquhoun, K. Culap, B. Jackson, F. Zatta, A. Rambaut, S. Jaconi, V. B. Sreenu, J. Nix, I. Zhang, R. F. Jarrett, W. G. Glass, M. Beltramello, K. Nomikou, M. Pizzuto, L. Tong, E. Cameroni, T. I. Croll, N. Johnson, J. Di Iulio, A. Wickenhagen, A. Ceschi, A. M. Harbison, D. Mair, P. Ferrari, K. Smollett, F. Sallusto, S. Carmichael, C. Garzoni, J. Nichols, M. Galli, J. Hughes, A. Riva, A. Ho, M. Schiuma, M. G. Semple, P. J. M. Openshaw, E. Fadda, J. K. Baillie, J. D. Chodera, S. J. Rihn, S. J. Lycett, H. W. Virgin, A. Telenti, D. Corti, D. L. Robertson, G. Snell, ISARIC4C Investigators, COVID-19 Genomics UK (COG-UK) Consortium, Circulating SARS-CoV-2 spike N439K variants maintain fitness while evading antibody-mediated immunity. *Cell* **184**, 1171–1187.e20 (2021). doi:10.1016/j.cell.2021.01.037 Medline

46. T. N. Starr, A. J. Greaney, S. K. Hilton, D. Ellis, K. H. D. Crawford, A. S. Dingens, M. J. Navarro, J. E. Bowen, M. A. Tortorici, A. C. Walls, N. P. King, D. Veesler, J. D. Bloom, Deep Mutational Scanning of SARS-CoV-2 Receptor Binding Domain Reveals Constraints on Folding and ACE2 Binding. *Cell* **182**, 1295–1310.e20 (2020). doi:10.1016/j.cell.2020.08.012 Medline

47. H. Shuai, J. F.-W. Chan, T. T.-T. Yuen, C. Yoon, J.-C. Hu, L. Wen, B. Hu, D. Yang, Y. Wang, Y. Hou, X. Huang, Y. Chai, C. C.-S. Chan, V. K.-M. Poon, L. Lu, R.-Q. Zhang, W.-M. Chan, J. D. Ip, A. W.-H. Chu, Y.-F. Hu, J.-P. Cai, K.-H. Chan, J. Zhou, S. Sridhar, B.-Z. Zhang, S. Yuan, A. J. Zhang, J.-D. Huang, K. K.-W. To, K.-Y. Yuen, H. Chu, Emerging SARS-CoV-2 variants expand species tropism to murines. *EBioMedicine* **73**, 103643 (2021). doi:10.1016/j.ebiom.2021.103643 Medline

48. T. Pan, R. Chen, X. He, Y. Yuan, X. Deng, R. Li, H. Yan, S. Yan, J. Liu, Y. Zhang, X. Zhang, F. Yu, M. Zhou, C. Ke, X. Ma, H. Zhang, Infection of wild-type mice by SARS-CoV-2 B.1.351 variant indicates a possible novel cross-species transmission route. *Signal Transduct. Target. Ther.* **6**, 420 (2021). doi:10.1038/s41392-021-00848-1 Medline

49. M. Hoffmann, N. Krüger, S. Schulz, A. Cossmann, C. Rocha, A. Kempf, I. Nehlmeier, L. Graichen, A.-S. Moldenhauer, M. S. Winkler, M. Lier, A. Dopfer-Jablonka, H.-M. Jäck, G. M. N. Behrens, S. Pöhlmann, The Omicron variant is highly resistant against antibody-mediated neutralization: Implications for control of the COVID-19 pandemic. *Cell* 10.1016/j.cell.2021.12.032 (2022). doi:10.1016/j.cell.2021.12.032

50. S. R. Leist, K. H. Dinnon 3rd, A. Schäfer, L. V. Tse, K. Okuda, Y. J. Hou, A. West, C. E. Edwards, W. Sanders, E. J. Fritch, K. L. Gully, T. Scobey, A. J. Brown, T. P. Sheahan, N. J. Moorman, R. C. Boucher, L. E. Gralinski, S. A. Montgomery, R. S. Baric, A

Mouse-Adapted SARS-CoV-2 Induces Acute Lung Injury and Mortality in Standard Laboratory Mice. *Cell* **183**, 1070–1085.e12 (2020). doi:10.1016/j.cell.2020.09.050 Medline

51. Y. Weisblum, F. Schmidt, F. Zhang, J. DaSilva, D. Poston, J. C. C. Lorenzi, F. Muecksch, M. Rutkowska, H.-H. Hoffmann, E. Michailidis, C. Gaebler, M. Agudelo, A. Cho, Z. Wang, A. Gazumyan, M. Cipolla, L. Luchsinger, C. D. Hillyer, M. Caskey, D. F. Robbiani, C. M. Rice, M. C. Nussenzweig, T. Hatziioannou, P. D. Bieniasz, Escape from neutralizing antibodies by SARS-CoV-2 spike protein variants. *eLife* **9**, e61312 (2020). doi:10.7554/eLife.61312 Medline

52. A. C. Walls, M. C. Miranda, A. Schäfer, M. N. Pham, A. Greaney, P. S. Arunachalam, M.-J. Navarro, M. A. Tortorici, K. Rogers, M. A. O’Connor, L. Shirreff, D. E. Ferrell, J. Bowen, N. Brunette, E. Kepl, S. K. Zepeda, T. Starr, C.-L. Hsieh, B. Fiala, S. Wrenn, D. Pettie, C. Sydeman, K. R. Sprouse, M. Johnson, A. Blackstone, R. Ravichandran, C. Ogohara, L. Carter, S. W. Tilles, R. Rappuoli, S. R. Leist, D. R. Martinez, M. Clark, R. Tisch, D. T. O’Hagan, R. Van Der Most, W. C. Van Voorhis, D. Corti, J. S. McLellan, H. Kleanthous, T. P. Sheahan, K. D. Smith, D. H. Fuller, F. Villinger, J. Bloom, B. Pulendran, R. S. Baric, N. P. King, D. Veesler, Elicitation of broadly protective sarbecovirus immunity by receptor-binding domain nanoparticle vaccines. *Cell* **184**, 5432–5447.e16 (2021). doi:10.1016/j.cell.2021.09.015 Medline

53. A. C. Walls, B. Fiala, A. Schäfer, S. Wrenn, M. N. Pham, M. Murphy, L. V. Tse, L. Shehata, M. A. O’Connor, C. Chen, M. J. Navarro, M. C. Miranda, D. Pettie, R. Ravichandran, J. C. Kraft, C. Ogohara, A. Palser, S. Chalk, E. C. Lee, K. Guerriero, E. Kepl, C. M. Chow, C. Sydeman, E. A. Hodge, B. Brown, J. T. Fuller, K. H. Dinnon 3rd, L. E. Gralinski, S. R. Leist, K. L. Gully, T. B. Lewis, M. Guttman, H. Y. Chu, K. K. Lee, D. H. Fuller, R. S. Baric, P. Kellam, L. Carter, M. Pepper, T. P. Sheahan, D. Veesler, N. P. King, Elicitation of Potent Neutralizing Antibody Responses by Designed Protein Nanoparticle Vaccines for SARS-CoV-2. *Cell* **183**, 1367–1382.e17 (2020). doi:10.1016/j.cell.2020.10.043 Medline

54. P. S. Arunachalam, A. C. Walls, N. Golden, C. Atyeo, S. Fischinger, C. Li, P. Aye, M. J. Navarro, L. Lai, V. V. Edara, K. Röltgen, K. Rogers, L. Shirreff, D. E. Ferrell, S. Wrenn, D. Pettie, J. C. Kraft, M. C. Miranda, E. Kepl, C. Sydeman, N. Brunette, M. Murphy, B. Fiala, L. Carter, A. G. White, M. Trisal, C.-L. Hsieh, K. Russell-Lodrigue, C. Monjure, J. Dufour, S. Spencer, L. Doyle-Meyers, R. P. Bohm, N. J. Maness, C. Roy, J. A. Plante, K. S. Plante, A. Zhu, M. J. Gorman, S. Shin, X. Shen, J. Fontenot, S. Gupta, D. T. O’Hagan, R. Van Der Most, R. Rappuoli, R. L. Coffman, D. Novack, J. S. McLellan, S. Subramaniam, D. Montefiori, S. D. Boyd, J. L. Flynn, G. Alter, F. Villinger, H. Kleanthous, J. Rappaport, M. S. Suthar, N. P. King, D. Veesler, B. Pulendran, Adjuvanting a subunit COVID-19 vaccine to induce protective immunity. *Nature* **594**, 253–258 (2021). doi:10.1038/s41586-021-03530-2 Medline

55. D. R. Martinez, A. Schäfer, S. R. Leist, G. De la Cruz, A. West, E. N. Atochina-Vasserman, L. C. Lindesmith, N. Pardi, R. Parks, M. Barr, D. Li, B. Yount, K. O. Saunders, D. Weissman, B. F. Haynes, S. A. Montgomery, R. S. Baric, Chimeric spike mRNA vaccines protect against Sarbecovirus challenge in mice. *Science* **373**, 991–998 (2021). doi:10.1126/science.abi4506 Medline

56. A. A. Cohen, P. N. P. Gnanapragasam, Y. E. Lee, P. R. Hoffman, S. Ou, L. M. Kakutani, J. R. Keeffe, H.-J. Wu, M. Howarth, A. P. West, C. O. Barnes, M. C. Nussenzweig, P. J. Bjorkman, Mosaic nanoparticles elicit cross-reactive immune responses to zoonotic coronaviruses in mice. *Science* **371**, 735–741 (2021). doi:10.1126/science.abf6840 Medline

57. C. Suloway, J. Pulokas, D. Fellmann, A. Cheng, F. Guerra, J. Quispe, S. Stagg, C. S. Potter, B. Carragher, Automated molecular microscopy: The new Leginon system. *J. Struct. Biol.* **151**, 41–60 (2005). doi:10.1016/j.jsb.2005.03.010 Medline

58. D. Tegunov, P. Cramer, Real-time cryo-electron microscopy data preprocessing with Warp. *Nat. Methods* **16**, 1146–1152 (2019). doi:10.1038/s41592-019-0580-y Medline

59. A. Punjani, J. L. Rubinstein, D. J. Fleet, M. A. Brubaker, cryoSPARC: Algorithms for rapid unsupervised cryo-EM structure determination. *Nat. Methods* **14**, 290–296 (2017). doi:10.1038/nmeth.4169 Medline

60. J. Zivanov, T. Nakane, B. O. Forsberg, D. Kimanius, W. J. Hagen, E. Lindahl, S. H. Scheres, New tools for automated high-resolution cryo-EM structure determination in RELION-3. *eLife* **7**, e42166 (2018). doi:10.7554/eLife.42166 Medline

61. S. H. Scheres, RELION: Implementation of a Bayesian approach to cryo-EM structure determination. *J. Struct. Biol.* **180**, 519–530 (2012). doi:10.1016/j.jsb.2012.09.006 Medline

62. A. Punjani, H. Zhang, D. J. Fleet, Non-uniform refinement: Adaptive regularization improves single-particle cryo-EM reconstruction. *Nat. Methods* **17**, 1214–1221 (2020). doi:10.1038/s41592-020-00990-8 Medline

63. J. Zivanov, T. Nakane, S. H. W. Scheres, A Bayesian approach to beam-induced motion correction in cryo-EM single-particle analysis. *IUCrJ* **6**, 5–17 (2019). doi:10.1107/S205225251801463X Medline

64. P. B. Rosenthal, R. Henderson, Optimal determination of particle orientation, absolute hand, and contrast loss in single-particle electron cryomicroscopy. *J. Mol. Biol.* **333**, 721–745 (2003). doi:10.1016/j.jmb.2003.07.013 Medline

65. S. Chen, G. McMullan, A. R. Faruqi, G. N. Murshudov, J. M. Short, S. H. Scheres, R. Henderson, High-resolution noise substitution to measure overfitting and validate resolution in 3D structure determination by single particle electron cryomicroscopy. *Ultramicroscopy* **135**, 24–35 (2013). doi:10.1016/j.ultramic.2013.06.004 Medline

66. E. F. Pettersen, T. D. Goddard, C. C. Huang, G. S. Couch, D. M. Greenblatt, E. C. Meng, T. E. Ferrin, UCSF Chimera—A visualization system for exploratory research and analysis. *J. Comput. Chem.* **25**, 1605–1612 (2004). doi:10.1002/jcc.20084 Medline

67. P. Emsley, B. Lohkamp, W. G. Scott, K. Cowtan, Features and development of Coot. *Acta Crystallogr. D* **66**, 486–501 (2010). doi:10.1107/S0907444910007493 Medline

68. R. Y. Wang, Y. Song, B. A. Barad, Y. Cheng, J. S. Fraser, F. DiMaio, Automated structure refinement of macromolecular assemblies from cryo-EM maps using Rosetta. *eLife* **5**, e17219 (2016). doi:10.7554/eLife.17219 Medline

69. B. Frenz, S. Rämisch, A. J. Borst, A. C. Walls, J. Adolf-Bryfogle, W. R. Schief, D. Veesler, F. DiMaio, Automatically Fixing Errors in Glycoprotein Structures with Rosetta. *Structure* **27**, 134–139.e3 (2019). doi:10.1016/j.str.2018.09.006 Medline

70. D. Liebschner, P. V. Afonine, M. L. Baker, G. Bunkóczi, V. B. Chen, T. I. Croll, B. Hintze, L. W. Hung, S. Jain, A. J. McCoy, N. W. Moriarty, R. D. Oeffner, B. K. Poon, M. G. Prisant, R. J. Read, J. S. Richardson, D. C. Richardson, M. D. Sammito, O. V. Sobolev, D. H. Stockwell, T. C. Terwilliger, A. G. Urzhumtsev, L. L. Videau, C. J. Williams, P. D. Adams, Macromolecular structure determination using X-rays, neutrons and electrons: Recent developments in Phenix. *Acta Crystallogr. D* **75**, 861–877 (2019). doi:10.1107/S2059798319011471 Medline

71. T. I. Croll, ISOLDE: A physically realistic environment for model building into low-resolution electron-density maps. *Acta Crystallogr. D* **74**, 519–530 (2018). doi:10.1107/S2059798318002425 Medline

72. V. B. Chen, W. B. Arendall 3rd, J. J. Headd, D. A. Keedy, R. M. Immormino, G. J. Kapral, L. W. Murray, J. S. Richardson, D. C. Richardson, MolProbity: All-atom structure validation for macromolecular crystallography. *Acta Crystallogr. D* **66**, 12–21 (2010). doi:10.1107/S0907444909042073 Medline

73. B. A. Barad, N. Echols, R. Y. Wang, Y. Cheng, F. DiMaio, P. D. Adams, J. S. Fraser, EMRinger: Side chain-directed model and map validation for 3D cryo-electron microscopy. *Nat. Methods* **12**, 943–946 (2015). doi:10.1038/nmeth.3541 Medline

74. J. Agirre, J. Iglesias-Fernández, C. Rovira, G. J. Davies, K. S. Wilson, K. D. Cowtan, Privateer: Software for the conformational validation of carbohydrate structures. *Nat. Struct. Mol. Biol.* **22**, 833–834 (2015). doi:10.1038/nsmb.3115 Medline

75. T. D. Goddard, C. C. Huang, E. C. Meng, E. F. Pettersen, G. S. Couch, J. H. Morris, T. E. Ferrin, UCSF ChimeraX: Meeting modern challenges in visualization and analysis. *Protein Sci.* **27**, 14–25 (2018). doi:10.1002/pro.3235 Medline

76. A. J. McCoy, R. W. Grosse-Kunstleve, P. D. Adams, M. D. Winn, L. C. Storoni, R. J. Read, Phaser crystallographic software. *J. Appl. Crystallogr.* **40**, 658–674 (2007). doi:10.1107/S0021889807021206 Medline

77. G. N. Murshudov, P. Skubák, A. A. Lebedev, N. S. Pannu, R. A. Steiner, R. A. Nicholls, M. D. Winn, F. Long, A. A. Vagin, REFMAC5 for the refinement of macromolecular crystal structures. *Acta Crystallogr. D* **67**, 355–367 (2011). doi:10.1107/S0907444911001314 Medline
